# Mesenchymal stem cells transplantation for perianal fistulas: a systematic review and meta-analysis of clinical trials

**DOI:** 10.1186/s13287-023-03331-6

**Published:** 2023-04-26

**Authors:** H. Wang, H. Y. Jiang, Y. X. Zhang, H. Y. Jin, B. Y. Fei, J. L. Jiang

**Affiliations:** 1 Scientific Research Center, China-Japan Union Hospital of Jilin University, Changchun, China; 2 Life Spring AKY Pharmaceuticals, Changchun, China; 3 Changchun University of Chinese Medicine, Changchun, China; 4 Department of Gastrointestinal Colorectal Surgery, China-Japan Union Hospital of Jilin University, Changchun, China

**Keywords:** Perianal fistulas, Mesenchymal stem cells transplantation, Clinical trials, Randomised controlled trial, Systematic review and meta-analysis, Healing rate, Efficacy and safety

## Abstract

**Background:**

Perianal fistulas, characterised as granulomatous inflammation of fistulas around the anal canal, are associated with significant morbidity resulting in a negative impact on quality of life and a tremendous burden to the healthcare system. Treatment of anal fistulas usually consists of anal surgery; however, results of closure rates are not satisfactory especially with complex perianal fistulas, after which many patients may suffer from anal incontinence. Recently, the administration of mesenchymal stem cells (MSCs) has shown promising efficacy. Herein, we aim to explore whether MSCs are effective for complex perianal fistulas and if they have either short-term, medium-term, long-term or over-long-term efficacy. Additionally, we want to elucidate whether factors such as drug dosage, MSC source, cell type, and disease aetiology influence treatment efficacy.

**Main body of the abstract:**

We searched four online databases and analysed data based on information within the clinical trials registry. The outcomes of eligible trials were analysed with Review Manager 5.4.1. Relative risk and related 95% confidence interval were calculated to compare the effect between the MSCs and control groups. In addition, the Cochrane risk of bias tool was applied to evaluate the bias risk of eligible studies. Meta-analyses showed that therapy with MSCs was superior to conventional treatment for complex perianal fistulas in short-, long- and over-long-term follow-up phases. However, there was no statistical difference in treatment efficacy in the medium term between the two methods. Subgroup meta-analyses showed factors including cell type, cell source and cell dosage were superior compared to the control, but there was no significant difference between different experimental groups of those factors. Besides, local MSCs therapy has shown more promising results for fistulas as a result of Crohn’s Disease (CD). Although we tend to maintain that MSCs therapy is effective for cryptoglandular fistulas equally, more studies are needed to confirm this conclusion in the future.

**Short conclusion:**

MSCs Transplantation could be a new therapeutic method for complex perianal fistulas of both cryptoglandular and CD origin showing high efficacy in the short-term to over-long-term phases, as well as high efficacy in sustained healing. The difference in cell types, cell sources and cell dosages did not influence MSCs’ efficacy.

**Supplementary Information:**

The online version contains supplementary material available at 10.1186/s13287-023-03331-6.

## Background

Perianal fistulas, characterised as granulomatous inflammation of fistulas around the anal canal, are associated with significant morbidity resulting in a negative impact on quality of life and a tremendous burden to the healthcare system [1–3]. As a common disorder, it is estimated to occur in 1.1–2.2 per 10,000 of the population [2, 4, 5]. Moreover, cryptoglandular infection and CD are considered predominant aetiologies, with a prevalence of 0.86 and 0.76 per 10,000 of the population, respectively [4]. Based on the fistula’s course, the scope of lesions and the number of external openings, perianal fistulas are categorised as simple or complex [6, 7]. Further, complex fistulas containing extrasphincteric, suprasphincteric, high intersphincteric, or high transsphincteric or with multiple external openings are associated with an abscess or with rectovaginal fistulas or anorectal stenosis [8, 9]. The more extensive normal tissue is involved, the more significantly the anal canal function has been impacted.

Treatment for anal fistulas usually consists of anal surgery, such as ligation of the intersphincteric fistula tract, fistulotomy or sphincteroplasty [6, 10]. Surgery results in closure rates ranging from 80 to 100% in the simple type of perianal fistulas; however, this does not provide a satisfactory result in the complex type, in which many patients suffer from faecal incontinence [11–14]. In addition, the high incidence rates of complications such as faecal incontinence and fistula recurrence significantly affect patients [15–17].

Recently, the administration of MSCs has shown promise in the treatment of complex perianal fistulas [18–20]. MSCs are characterised by self-renewal and multi-differentiation ability [21]. Furthermore, they can secrete cytokines through autocrine or paracrine modes in a morbid environment to promote re-epithelialisation and blood vessel regeneration [22, 23]. Currently, MSCs transplantation for complex perianal fistula treatment has entered phase II and phase III clinical trials with stellar prospects. Therefore, we aim to discuss the efficacy and safety of MSCs transplantation for perianal fistulas in this article.

## Materials and methods

This study was performed in accordance with the methodology of the Preferred Reporting Items for Systematic Reviews and Meta-Analysis guidelines [24].

### Search strategy

A comprehensive search of PubMed, Embase, Cochrane library database and the US ClinicalTrials.gov was performed for all relevant records before 15 May 2022.

Medical subject headings or Emtree terms were combined with Boolean operators and used to search the databases. All searches were performed without language restriction up to 15th May 2022. The details of the search strategy are presented in appendix 1.

### Study selection

Inclusion criteria: (1) clinical trials; (2) randomised controlled trials (RCTs); (3) complex perianal fistulas: cryptoglandular origin or associated with CD; (4) interventions of experimental group: local management with MSCs alone or added on to current medical treatment; interventions of control group: placebo or conventional treatment (fibrin glue, surgery, etc.); (5) outcomes: healing rates (HR); (6) language: English.

Exclusion criteria: (1) non-human studies; (2) type of articles: one-arm clinical trials, reviews, case reports, meta-analysis, records of meetings, conference abstracts, and letters to the editor; (3) studies for which the full text cannot be obtained; (4) systemic use of MSCs; (5) healing rate was not reported.

Two reviewers assessed the titles and abstracts of studies independently. The full texts of studies that appeared to meet the inclusion criteria or with insufficient information in the title/abstract were scanned to determine which could be included. Disagreements were solved by discussion.

### Data extraction

Two investigators (H Wang, HY Jiang) carried out data extraction independently. The following terms were collected from eligible studies: (1) first author; (2) publication date and experimental location; (3) demographic characteristics of participants (total number of every trial, the number of each group, age, and gender); (3) perianal fistulas type; (4) type, source, and dosage of MSCs; (5) interventions for the experimental and control groups; and (6) length of follow-up and HR.

### Study quality

The Cochrane risk of bias tool was applied to evaluate bias risk of eligible studies by two investigators. Six aspects were assessed for each study: selection bias, performance bias, detection bias, attrition bias, reporting bias and other biases. Each item was classified as high risk, low risk or unclear.

When reviewing the full texts to extract data, our investigators observed that one clinical trial with a series of HR data and follow-up phases was associated with several publications. Our work group members then consistently regarded the data from the same trial as a unit to analyse, meaning we summarised data based on the trials rather than the publications.

### Statistical analysis

The HR of participants was considered as the main endpoint. We analysed and managed all data through Review Manager 5.4.1. Relative risk (RR) and related 95% confidence intervals (CI) were calculated to compare the effect between the MSCs and control groups. Statistical heterogeneity between the eligible studies was evaluated by the *I*^2^ test (*I*^2^ value < 0% indicating low heterogeneity). When *I*^2^ < 50%, a fixed-effects model was chosen. To identify heterogeneity, sensitivity analysis with an omission of one study at a time was performed. The odd clinical trial was omitted to lower heterogeneity, and then, a fixed-effects model was implemented to the remaining data. Only when the number of eligible studies was too small to omit, a random model was chosen. Statistical significance was considered at a P-value of < 0.05. The funnel plot was used to test publication bias.

## Results

### Literature search and quality assessment

**Fig. 1:**
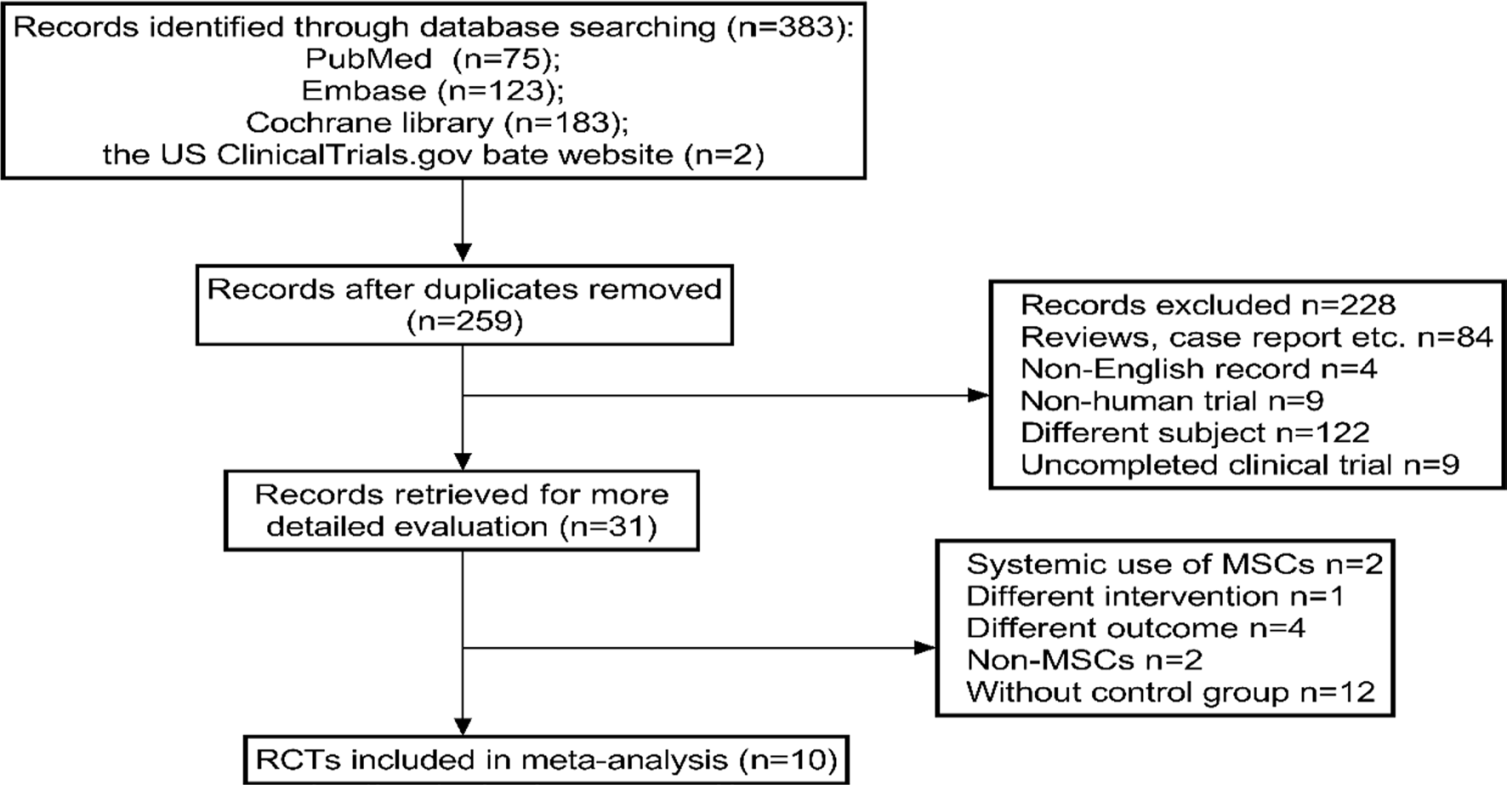
Flow diagram study selection

In total, 383 records were obtained from the computerised search. After preliminary screening and scanning of the full texts, 10 publications were admitted into our study. Figure 1 shows the detailed literature screening process. The 10 publications were from six clinical trials. Table 1 shows the relationships among the eligible publications. Those publications reported HR of different follow-up phases in clinical trials. The results of two publications [18, 25] from clinical trial 1, one publication [26] from clinical trial 2, two publications [27, 28] from clinical trial 3, three publications [29–31] from clinical trial 4, one publication [32] from clinical trial 5, and one publication [33] from clinical trial 6. The length of follow-up in the six trials ranged from 8 weeks to 4 years, resulting in HR of different phases, which could be used to analyse the efficacy of MSCs therapy.

**Table 1:** The relationships among the eligible 10 publications

Clinical trial no.	First author	Publication date	Location	Follow-up phase (after-treatment)
1	Garcia-Olmo, D. [18]	2009	Spain	8 weeks
	Hector, G. [25]	2012	Spain	(1) 1 year
				(2) 40 months
2	Herreros, M. D. [26]	2012	Spain	(1) 12 weeks
				(2) 24/26 weeks
				(3) 1 year
3	Molendijk, I. [27]	2015	Netherlands	(1) 12 weeks
				(2) 24 weeks
	Barnhoorn, M. C. [28]	2020	Netherlands	4 years
	Panés, J. [29]	2016	Seven European countries and Israel	24 weeks
4	Panés, J. [30]	2018	Seven European countries and Israel	52 weeks
	Garcia-Olmo, D. [31]	2022	Seven European countries and Israel	104 weeks
5	Garcia-Arranz, M. [32]	2020	Spain	(1) 16 weeks
				(2) 52 weeks
				(3) 2 years
6	Zhou, C. [33]	2020	China	(1) 3 months
				(2) 6 months
				(3) 12 months

^a^To avoid bias, the follow-up phase was not adjusted by the same unit like week or month and just presented as 10 publications recording

**Fig. 2:**
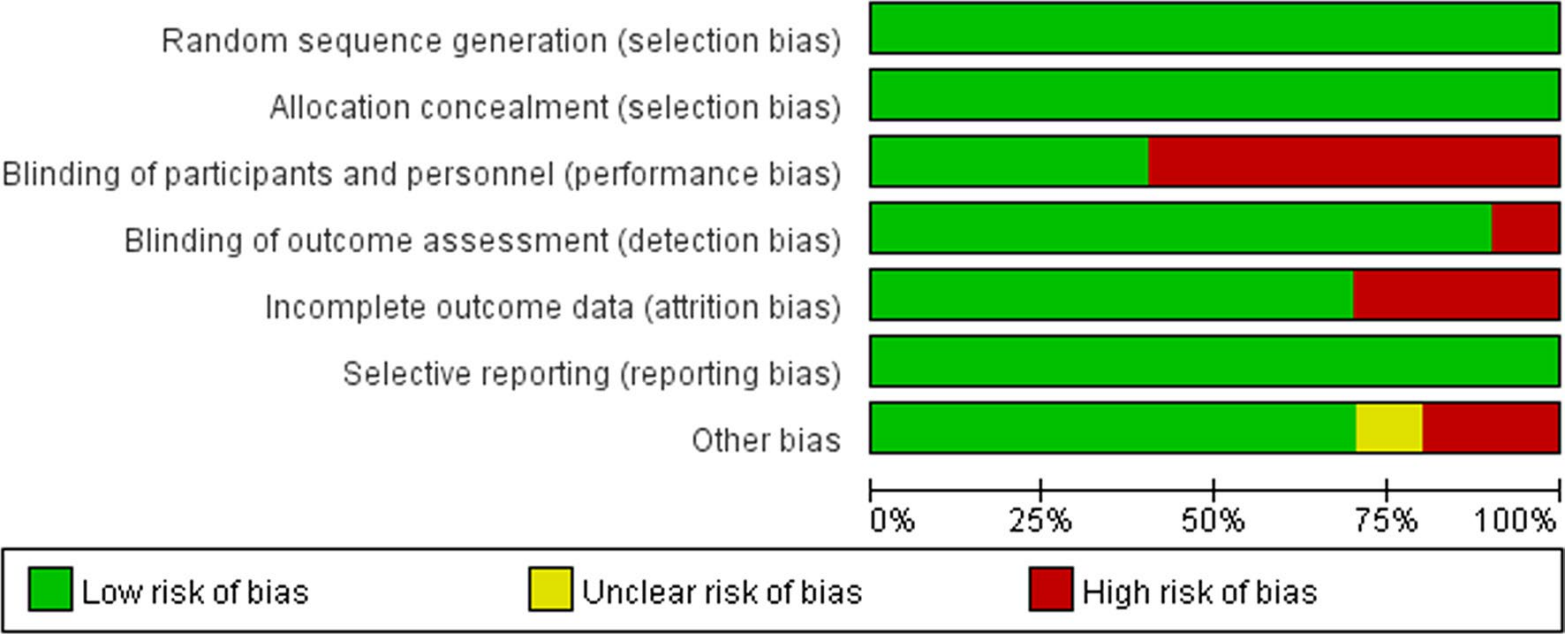
Risk of bias graph of eligible publications

**Fig. 3:**
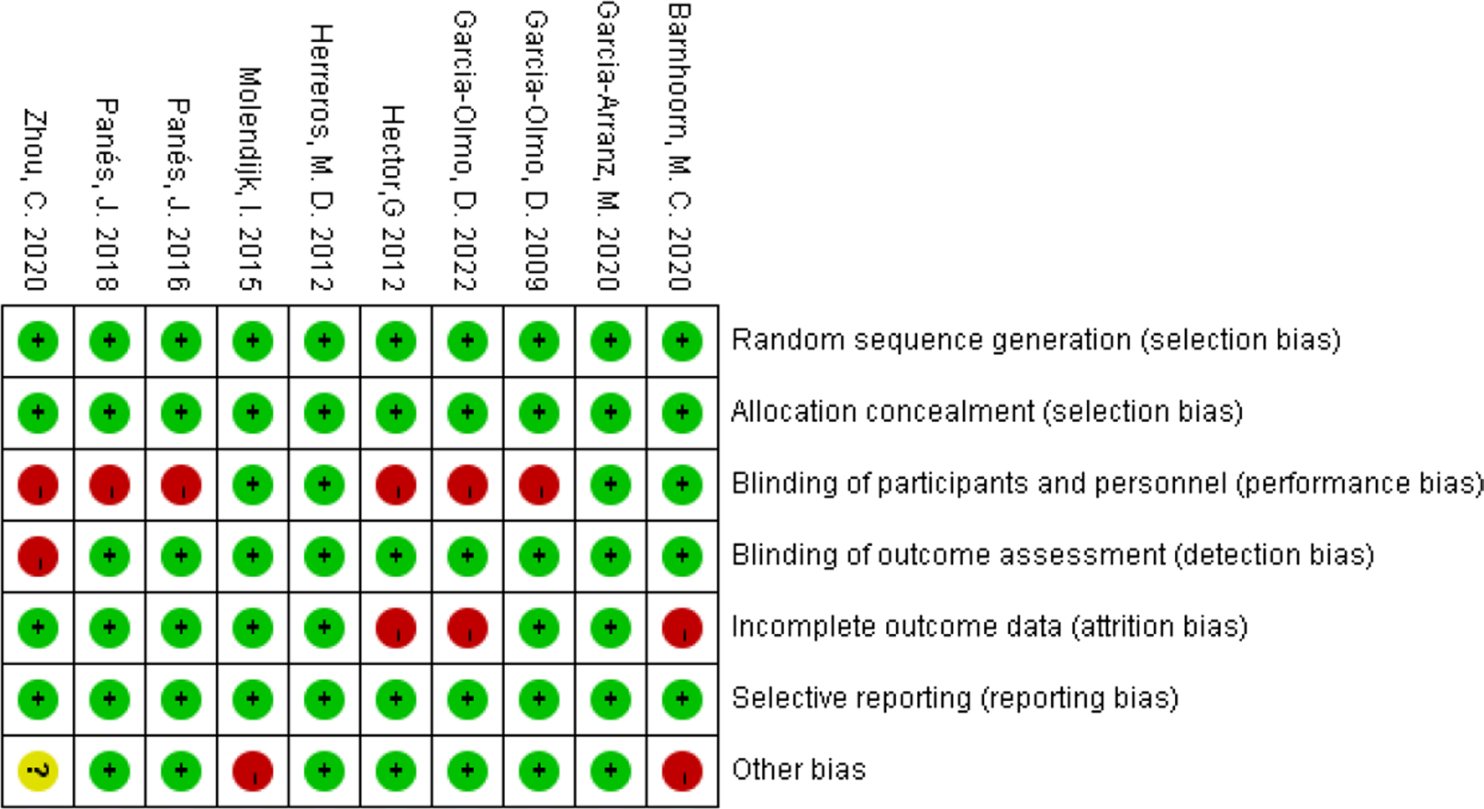
Risk of bias summary of eligible publications

The risk of bias in the eligible publications is listed in Figs. 2 and 3. All publications were detected as low risk of bias in random sequence generation, allocation concealment, and selective reporting. Meanwhile, a low risk of bias was also detected in the blinding of outcome assessment, incomplete outcome data and other biases among a majority of the eligible publications. A high risk of bias was mostly detected in blinding of participants and personnel.

### Basic demographics and clinical characteristics

The six eligible trials contained 10 publications, and each publication met the criteria. The perianal fistulas of every participant were of cryptoglandular origin or were associated with CD: clinical trial 1 contained two types of participants; clinical trials 2 and 5 only contained the former type of participants; and clinical trials 3, 4, and 6 only contained the last type. The number of cases included in the six trials ranged from 21 to 212 and contained 487 in total. All participants were > 18 years old with an unequal course of complex perianal fistulas, and more men than women were in admittance to these clinical trials.

Five clinical trials (clinical trials 1, 2, 4, 5, and 6) used adipose-derived MSCs (ASCs), and one (clinical trial 3) used bone marrow-derived MSCs (BSCs) in the experimental group. Four clinical trials (clinical trials 1, 2, 5, and 6) used autologous MSCs, and two (clinical trials 3 and 4) used allogeneic MSCs. The dosage of MSCs applied in the trials ranged from 1 × 10^7^ to 10 × 10^7^ per participant, and the maximum dose was applied in clinical trial 6, while the minimum dose was applied in clinical trial 3. In three trials (clinical trials 1, 2, and 5), participants who were defined as unhealed after the first-time treatment would receive the second time treatment. Unhealed participants would receive a double dose of the first-time intervention in the second treatment in clinical trials 1 and 2. The same dose of intervention would be used to treat in the second treatment in clinical trial 5.

Patients who required further surgical procedures in the follow-up phase were considered treatment failures. The basic demographics and clinical characteristics of eligible trials are shown in Tables 2 and 3. No statistical heterogeneity of basic demographics existed between the experimental and control groups in every clinical trial. We demonstrated the experimental procedures from recruitment to the last follow-up time of each eligible trial. Additional file 1: Figs. S1–S6 in Appendix 2 show the flow diagram of the six clinical trials.

**Table 2:** Basic demographics of six clinical trails

Clinical trial no.	Type of perianal fistulas	N (experimental group vs. control group)	Age (mean ± SD)		Gender (male:female)	
			Experimental group	Control group	Experimental group	Control group
1	(1). Cryptoglandular (2). CD	49 (24 vs. 25)	42.64 ± 10.93	43.99 ± 8.97	10:14	14:11
2	Cryptoglandular	183 (64, 60 vs. 59)	49.78 ± 11.39	50.85 ± 12.51	47:17	44:15
			47.27 ± 12.27		36:24	
3	CD	21 (5, 5, 5 vs. 6)	40.4 ± 10.29	37.3 ± 8.82	4:1	3:3
			40.8 ± 3.80		4:1	
			33.4 ± 11.63		1:4	
4	CD	212 (107 vs. 105)	39.0 ± 13.1	37.6 ± 13.1	60:47	56:49
5	Cryptoglandular	44 (23 vs. 21)	50.10 ± 10.7	50.86 ± 9.64	16:7	14:7
6	CD	22 (11 vs. 11)	24.4 ± 5.0	24.9 ± 5.4	11:0	10:1

**Table 3:** The intervention and outcomes of six clinical trails

Clinical trial no.	Cell type and source	Intervention	
		Experimental group (fixed cell dose)	Control group
*(a)*			
1	ASCs autologous	First: 2 × 10^7^ + fibrin glue	First: fibrin glue
		Second: double dose	Second: double dose
2	ASCs autologous	First: 2 × 10^7^, 2 × 10^7^ + fibrin glue	First: fibrin glue
		Second: double dose	Second: double dose
3	BSCs allogenic	Group 1: 1 × 10^7^	Saline solution (2.5–5 ml)
		Group 2: 3 × 10^7^	
		Group 3: 9 × 10^7^	
4	ASCs allogenic	12 × 10^7^	Saline solution: 24 ml
5	ASCs autologous	First: 10 × 10^7^ + fibrin glue	First: Fibrin glue (2–5 ml)
		Second: 10 × 10^7^ + fibrin glue	Second: Fibrin glue (2–5 ml)
6	ASCs autologous	(142.3 ± 45.7) × 10^6^	Surgery

Outcome assessment	Results (healed)			
	≤ 3 months	> 3 months, ≤ 6 months	1 year (48 ~ 52 weeks)	> 1 year
*(b)*				
Reepithelialisation; Reepithelialisation + MRI	17/24 vs. 3/25	/	15/24 vs. 3/25	7/21 vs. 2/13
Reepithelialisation + MRI	17/64, 23/60 vs. 9/59	25/64, 26/60 vs. 22/59	24/42, 22/42 vs. 19/51	/
Reepithelialisation + MRI	2/5, 4/5, 1/5 vs. 2/6	4/5, 4/5, 1/5 vs. 2/6	/	3/4, 4/4, 1/5 vs. 0/3
Reepithelialisation + MRI	/	53/103 vs. 36/101	58/103 vs. 39/101	14/25 vs. 6/15
Reepithelialisation + MRI	/	7/23 vs. 9/21	11/20 vs. 12/19	10/20 vs. 5/19
Reepithelialisation + MRI/ERUS	10/11 vs. 5/11	8/11 vs. 6/11	7/11 vs. 6/11	/

### Efficacy of MSCs for complex perianal fistulas

HR was the primary endpoint in our meta-analysis. Healing was defined as the complete re-epithelisation of external openings and/or the absence of drainage through those openings by magnetic resonance imaging.

#### Meta 1: Efficacy of MSCs for complex perianal fistulas in short-term follow-up phase (≥ 3 months after treatment)

**Fig. 4:**
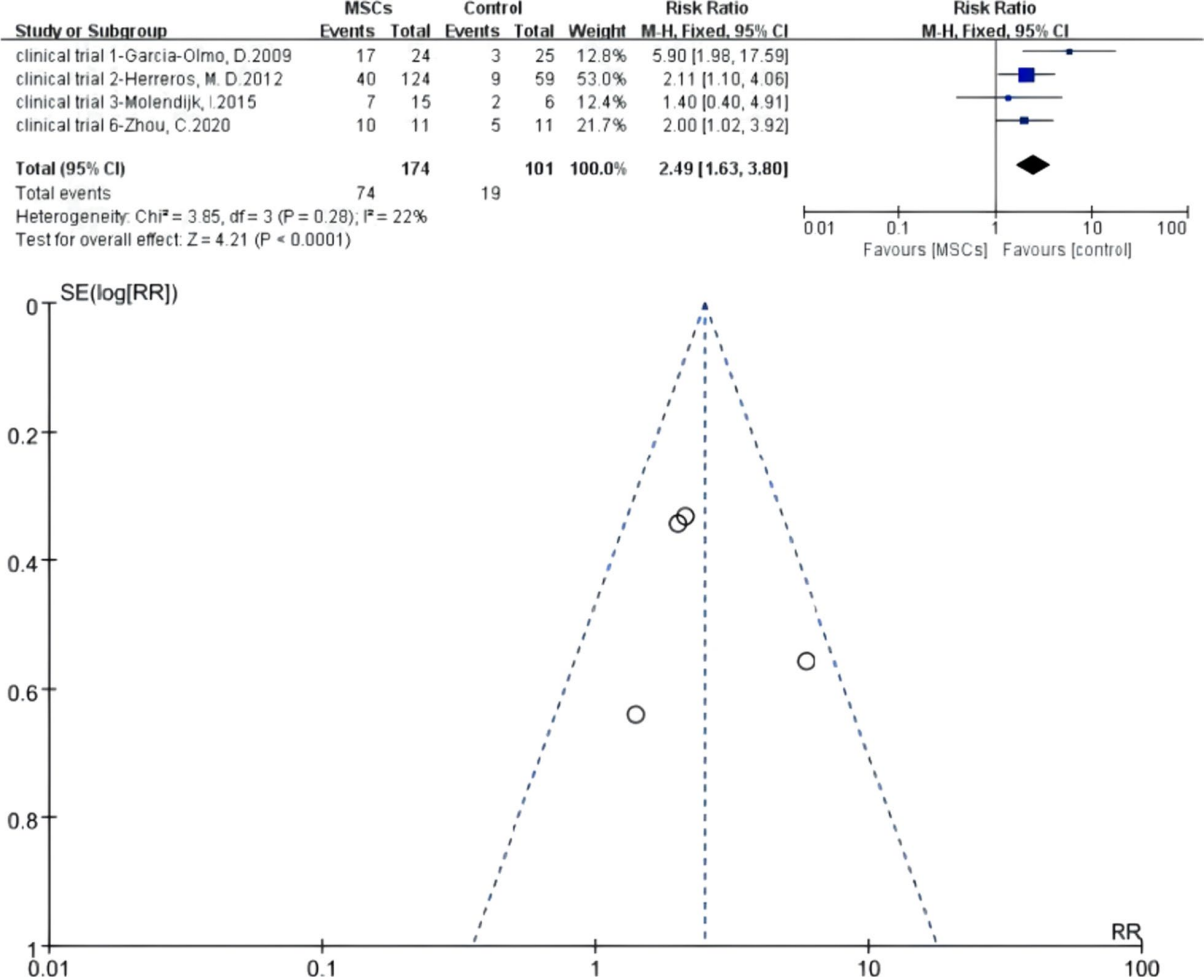
Efficacy of MSCs group versus the control group in short-term follow-up phase (forest plot and funnel plot)

Four RCTs were eligible. There were 174 versus 101 participants in the MSCs group (MSCs/MSCs + fibrin glue) and control group (fibrin glue/saline solution/surgery). *I*^2^ test (*I*^2^ = 22% < 50%) indicated low heterogeneity. A fixed-effects model was applied; *z* = 4.21 and *P* < 0.0001 indicated that the efficacy of MSCs for complex perianal fistulas was superior to traditional treatment (RR = 2.49; 95% CI 1.63, 3.80). The funnel plot showed that no publication bias existed (Fig. 4).

#### Meta 2: Efficacy of MSCs for complex perianal fistulas in mediate-term follow-up phase (≤ 6 months after treatment)

**Fig. 5:**
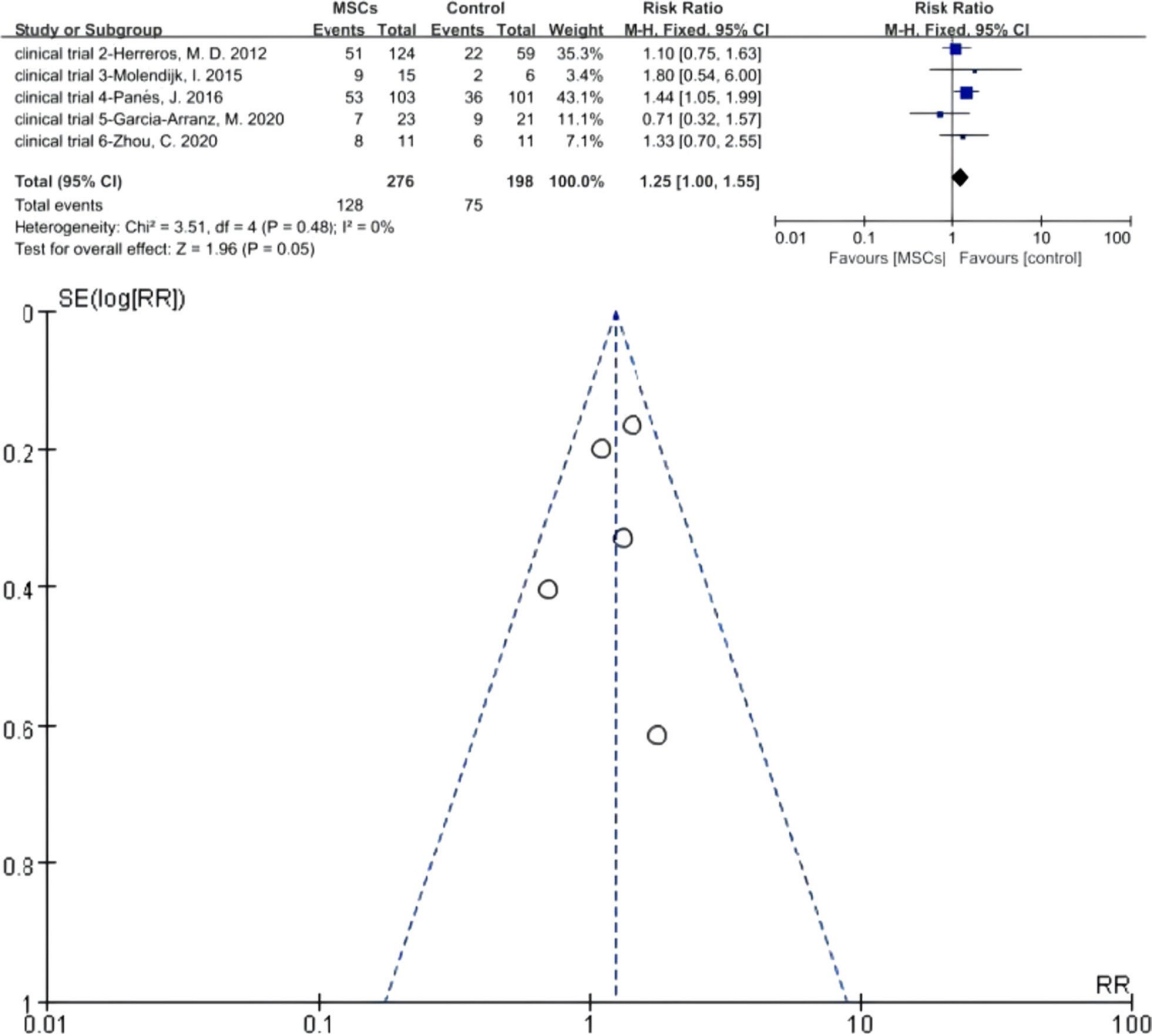
Efficacy of MSCs group versus the control group in mediate-term follow-up phase (forest plot and funnel plot)

Five RCTs were eligible. There were 276 versus 198 participants in the MSCs group (MSCs/MSCs + fibrin glue) and control group (fibrin glue/saline solution/surgery), respectively. *I*^2^ test (*I*^2^ = 0% < 50%) indicated low heterogeneity. A fixed-effects model was applied; *z* = 1.96 and *P* = 0.05 indicated no statistical significance of efficacy between the two groups (RR = 1.25; 95% CI 1.00, 1.55). The funnel plot showed that no publication bias existed (Fig. 5).

#### Meta 3: Efficacy of MSCs for complex perianal fistulas in long-term follow-up phase (approximately 1 year after treatment)

**Fig. 6:**
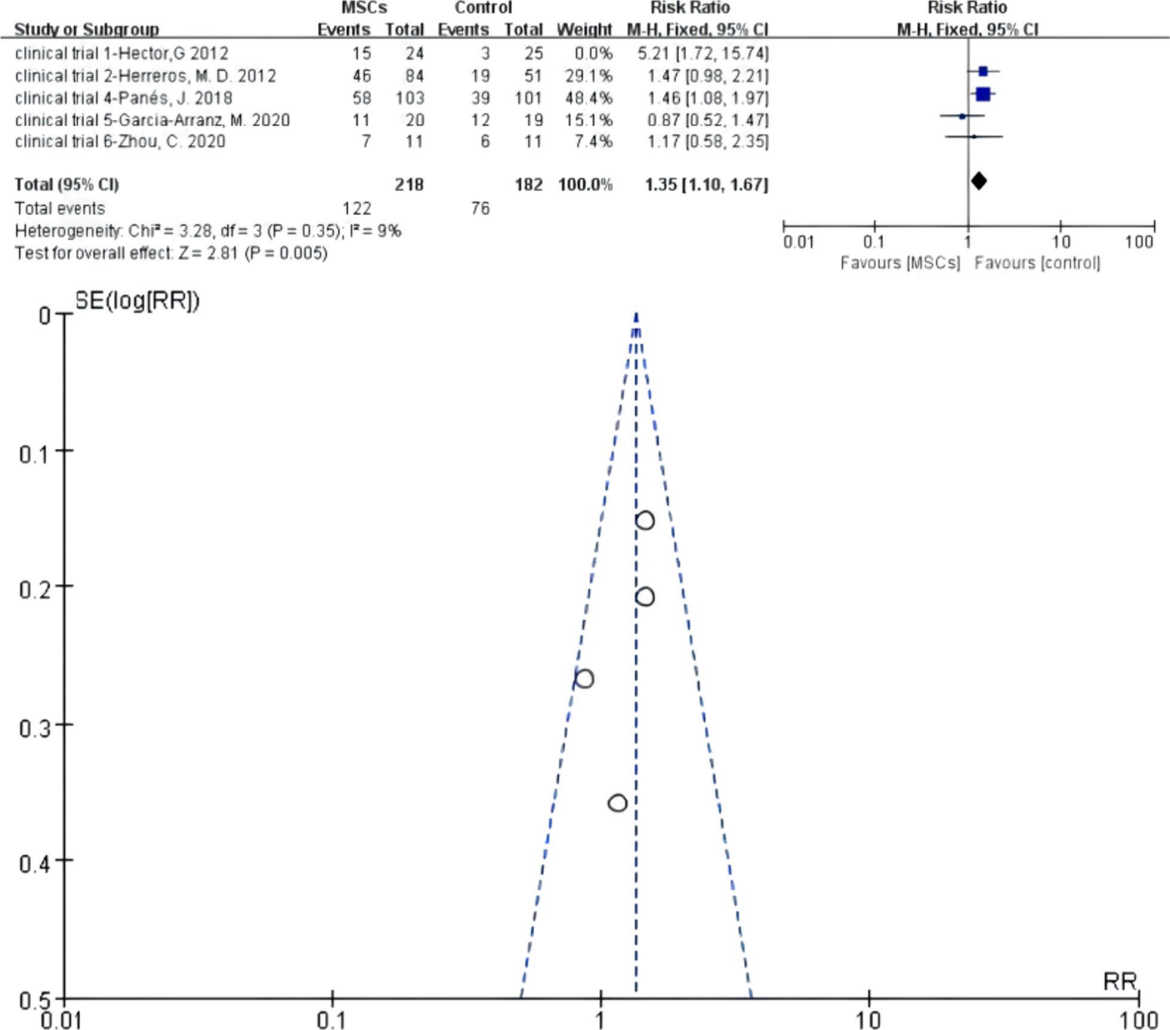
Efficacy of MSCs group versus the control group in long-term follow-up phase (forest plot and funnel plot)

Five RCTs were eligible, and heterogeneity existed when they were all adopted in the meta-analysis (*I*^2^ > 50%). A sensitivity analysis with an omission of one study at a time indicated that clinical trial 1—Hector [25] and clinical trial 5—Garcia-Arranz [32] would increase the heterogeneity, respectively. If any of these two was omitted, the heterogeneity decreased (*I*^2^ < 50%). However, the funnel plot showed that if the omitted one was clinical trial 5—Garcia-Arranz [32], there was still one study that resulted in publication bias. Hence, we finally omitted clinical trial 1—Hector [25], and then, the *I*^2^ test (*I*^2^ = 9% < 50%) indicated low heterogeneity. Of the remaining trials, there were 218 versus 182 participants in the MSCs group (MSCs/MSCs + fibrin glue) and 1 control group (fibrin glue/saline solution/surgery), respectively. A fixed-effects model was applied; *z* = 2.81 and *P* = 0.005 < 0.05 indicated that the efficacy of MSCs for complex perianal fistulas was superior to that of traditional treatment (RR = 1.35; 95% CI 1.10, 1.67) (Fig. 6).

#### Meta 4: Efficacy of MSCs for complex perianal fistulas in over long-term follow-up phase (> 1 year after treatment)

**Fig. 7:**
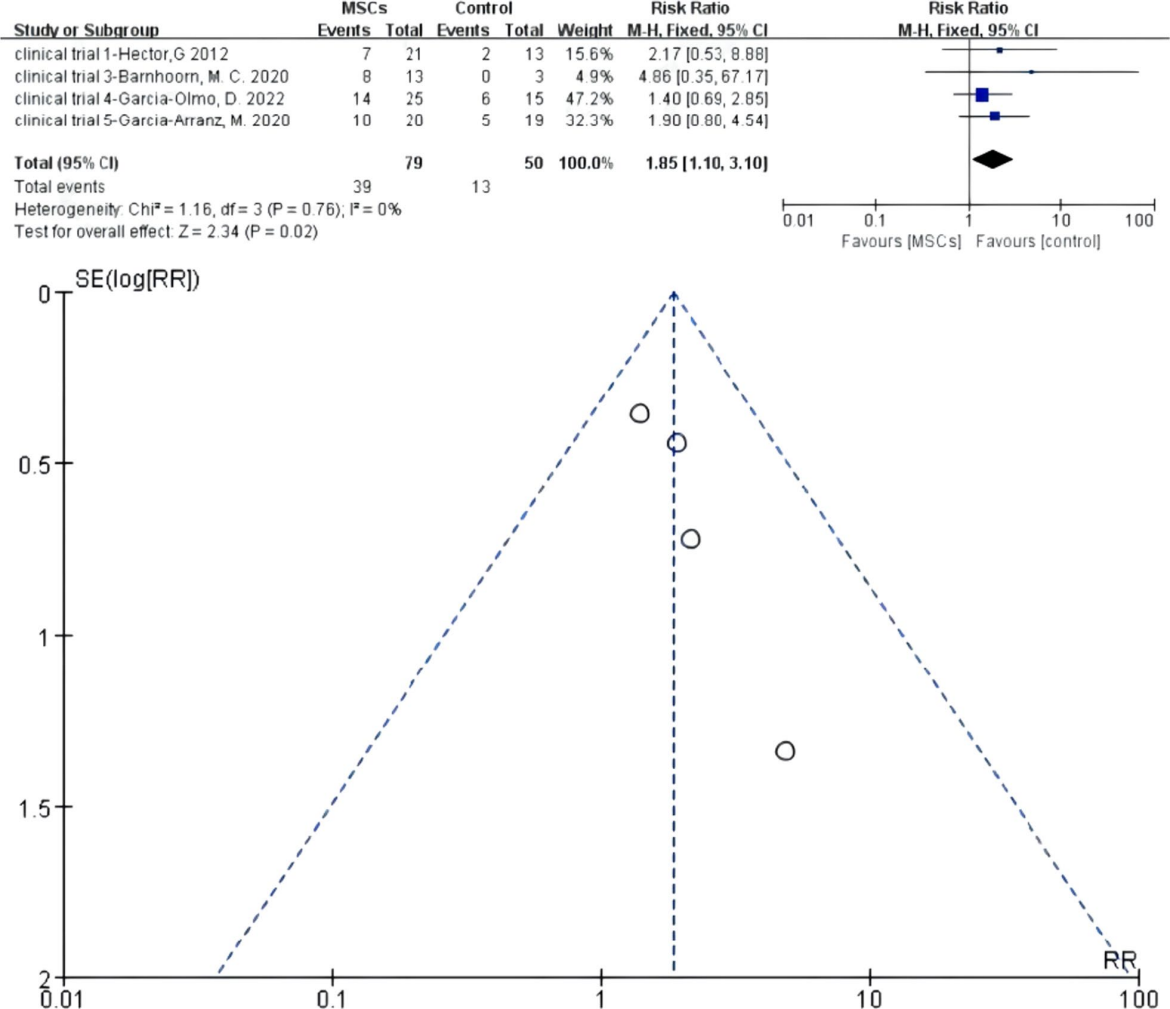
Efficacy of MSCs group versus the control group in over long-term follow-up phase (forest plot and funnel plot)

Four RCTs were eligible. There were 79 versus 50 participants in the MSCs group (MSCs/MSCs + fibrin glue) and control group (fibrin glue/saline solution), respectively. *I*^2^ test (*I*^2^ = 0% < 50%) indicated low heterogeneity. A fixed-effects model was applied; *z* = 2.34 and *P* = 0.02 < 0.05 indicated that the efficacy of MSCs for complex perianal fistulas was superior to that of traditional treatment (RR = 1.85; 95% CI 1.10, 3.10). The funnel plot showed that no publication bias existed (Fig. 7).

#### Meta 5: MSCs for different types of complex perianal fistulas (CD or cryptoglandular)

**Fig. 8:**
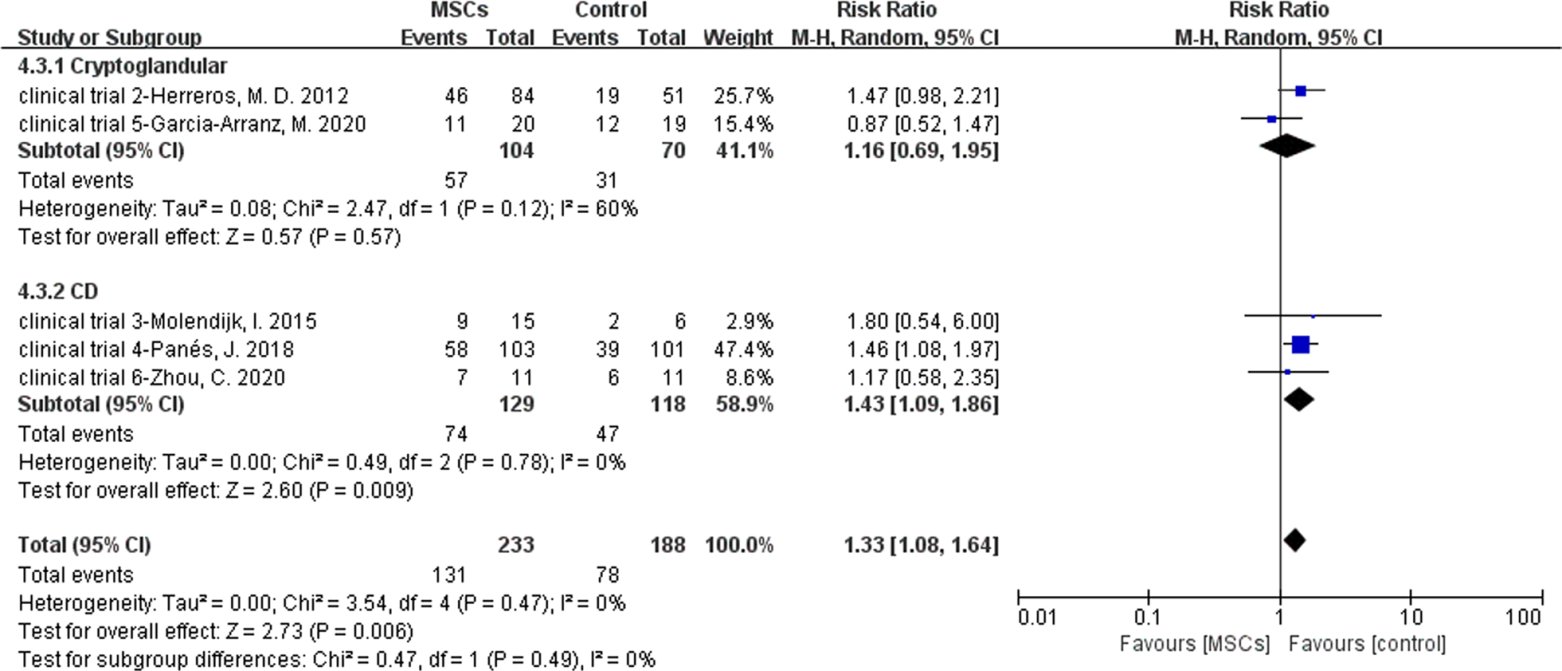
Efficacy of MSCs for different types of cryptoglandular or CD complex perianal fistulas (forest plot)

Two of six RCTs reported MSCs for the treatment of cryptoglandular infection fistulas. There were 104 versus 70 participants in the MSCs group (MSCs/MSCs + fibrin glue) and control group (fibrin glue/saline solution). Four of six RCTs reported MSCs for the treatment of CD fistulas. There were 129 versus 118 participants in the MSCs group and control group. Because there were only two studies in cryptoglandular subgroup and *I*^2^ = 60%, we could not omit anyone. As a result, a random model was applied. In cryptoglandular subgroup, RR = 1.16, 95% CI (0.69, 1.95) and *Z* = 0.57, *P* = 0.57 indicated no statistical significance of this subgroup even though the HR of MSCs group was superior compared to the control group (54.81% versus 44.29%). In CD group, the HR of MSCs group was also superior compared to the control group (57.36% versus 39.83%; RR = 1.43, 95% CI 1.09, 1.8; *Z* = 2.60, *P* = 0.009) (Fig. 8).

#### Meta 6: Different cell types of MSCs for the treatment of complex perianal fistulas

**Fig. 9:**
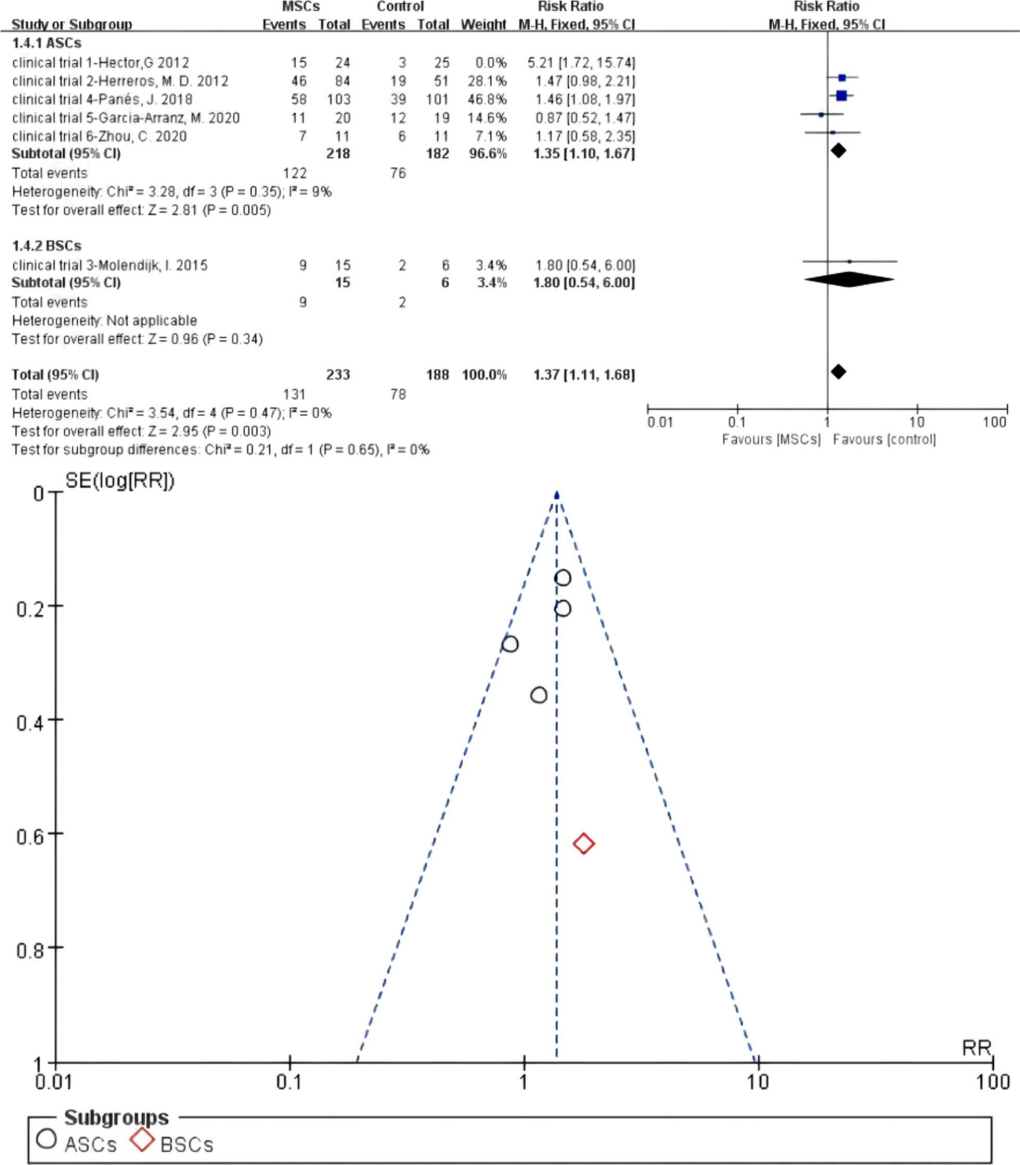
Forest plot of different cell types of MSCs for the treatment of complex perianal fistulas (forest plot and funnel plot)

Five of six RCTs reported ASCs for the treatment of fistulas. There were 218 versus 182 participants in the MSCs group (MSCs/MSCs + fibrin glue) and control group (fibrin glue/saline solution). Heterogeneity existed when they were all adopted in the meta-analysis (*I*^2^ > 50%). A sensitivity analysis with an omission of one study at a time indicated that clinical trial 1—Hector [25] would increase the heterogeneity, so we omitted this study. Then, *I*^2^ test (*I*^2^ = 9% < 50%) indicated low heterogeneity, and a fixed-effects model was applied. One study reported BSCs for the treatment of fistulas of 15 versus 6 participants in the MSCs group and control group, respectively. The pooled analysis showed that the ASCs subgroup (55.96% versus 41.76%; RR = 1.35, 95% CI 1.10, 1.67; *Z* = 2.81, *P* = 0.005) and BSCs subgroup (60.00% versus 33.33%; RR = 1.80, 95% CI 0.54, 6.00) can obtain higher HR than the control group. For the total events, *I*^2^ test (*I*^2^ = 0% < 50%) indicated low heterogeneity (Fig. 9).

#### Meta 7: Different sources of MSCs for the treatment of complex perianal fistulas

**Fig. 10:**
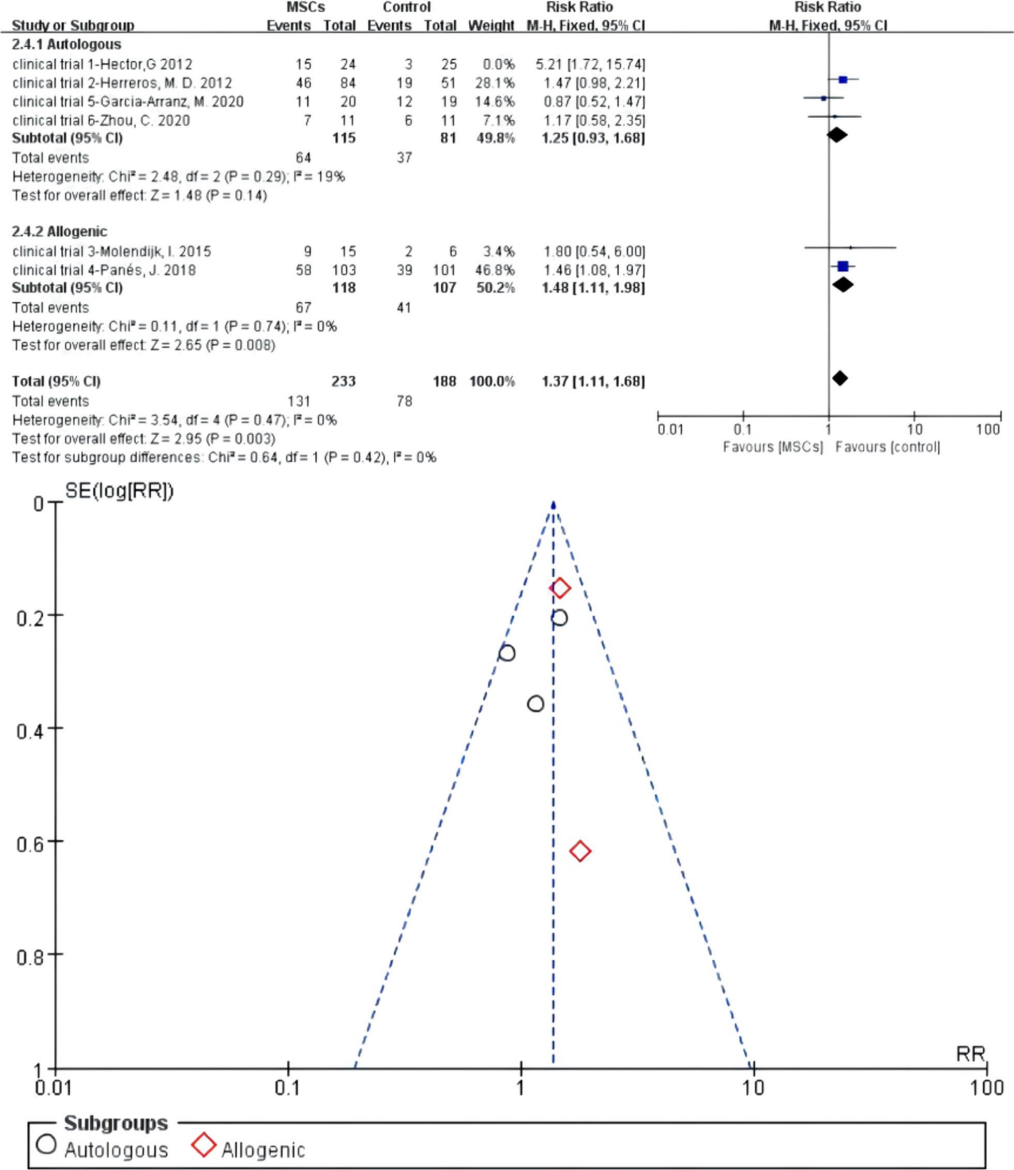
Different sources of MSCs for the treatment of complex perianal fistulas (forest plot and funnel plot)

Four of six RCTs reported autologous MSCs for the treatment of fistulas. There were 115 versus 81 participants in the autologous MSCs subgroup (MSCs/MSCs + fibrin glue) and control group (fibrin glue/saline solution). Heterogeneity existed when they were all adopted in the meta-analysis (*I*^2^ > 50%). A sensitivity analysis with an omission of one study at a time indicated that clinical trial 1—Hector [25] would increase the heterogeneity, so we omitted this study. The *I*^2^ test (*I*^2^ = 19% < 50%) indicated low heterogeneity. Two studies reported allogenic MSCs for the treatment of fistulas, and there were 118 versus 107 participants in the allogenic MSCs subgroup and control group, respectively. *I*^2^ test of allogenic MSCs subgroup (*I*^2^ = 0% < 50%) indicated low heterogeneity, and a fixed-effects model was applied. The pooled analysis showed that autologous MSCs subgroup (55.65% versus 45.68%; RR = 1.25, 95% CI 0.93, 1.68; *Z* = 1.48, *P* = 0.14) and allogenic MSCs subgroup (56.78% versus 38.32%; RR = 1.48, 95% CI 1.11, 1.98) both can obtain higher HR than the control group. For the total events, *I*^2^ test (*I*^2^ = 0% < 50%) indicated low heterogeneity (Fig. 10).

#### Meta 8: Different dosage of MSCs for the treatment of complex perianal fistulas

**Fig. 11:**
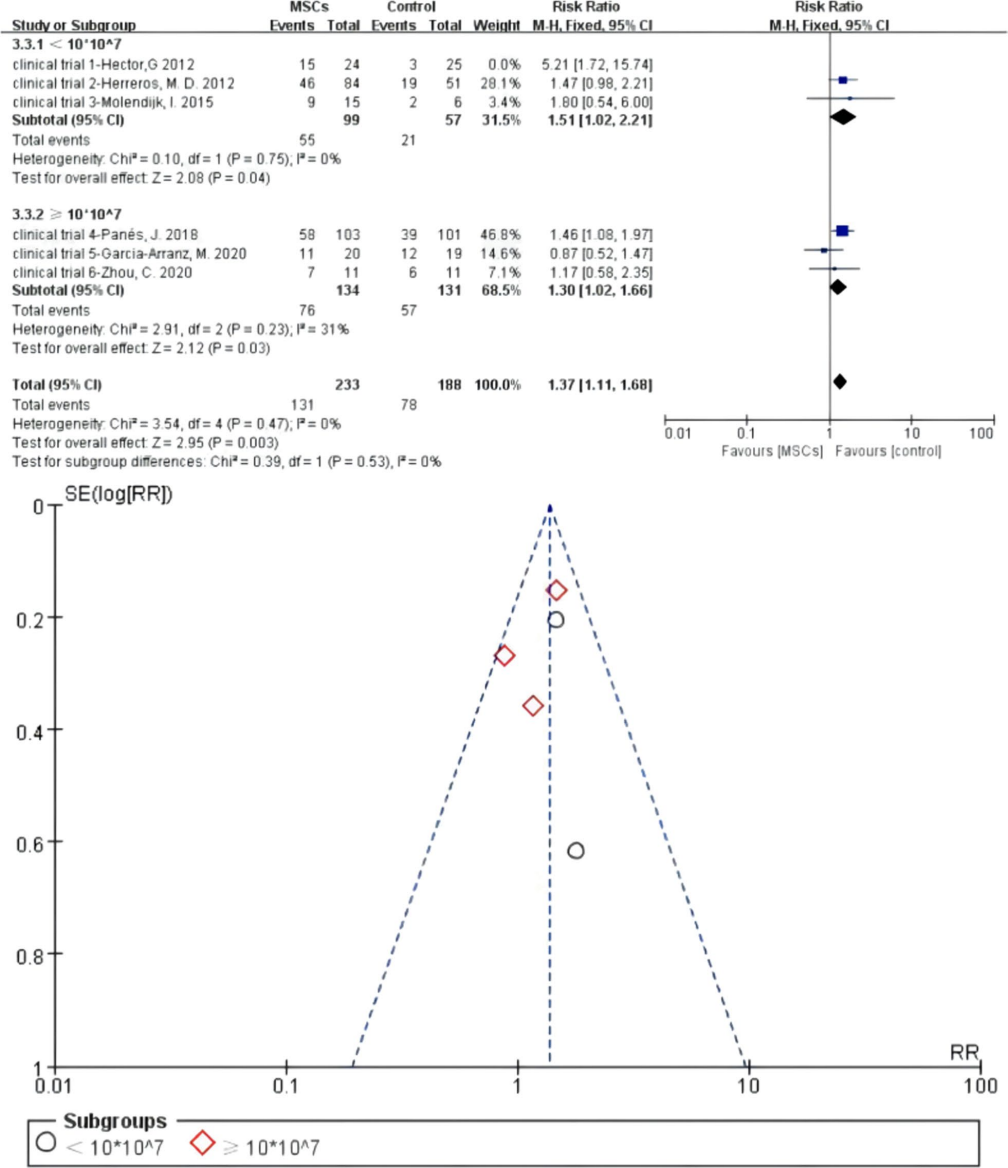
Different dosage of MSCs for the treatment of complex perianal fistulas (forest plot and funnel plot)

Bounded by 10 × 10^7^ total cell dosage in the full course of treatment, three of six RCTs reported low dosage of MSCs (< 10 × 10^7^) for the treatment of fistulas. There were 99 versus 57 participants in the MSCs subgroup (MSCs/MSCs + fibrin glue) and control group (fibrin glue/saline solution). Heterogeneity existed when they were all adopted in the meta-analysis (*I*^2^ > 50%). A sensitivity analysis with an omission of one study at a time indicated that clinical trial 1—Hector [25] would increase the heterogeneity, so we omitted this study. Then, *I*^2^ test (*I*^2^ = 0% < 50%) indicated low heterogeneity. Three studies reported high dosage of MSCs (≥ 10 × 10^7^) for the treatment of fistulas and 134 versus 131 participants in the MSCs subgroup and control group. *I*^2^ test of allogenic MSCs subgroup (*I*^2^ = 31% < 50%) indicated low heterogeneity. A fixed-effects model was applied. The pooled analysis showed that low dosage MSCs subgroup (55.56% versus 36.84%; RR = 1.51, 95% CI 1.02, 2.21; *Z* = 2.08, *P* = 0.04) and high dosage MSCs subgroup (56.72% versus 43.51%; RR = 1.30, 95% CI 1.02, 1.66) can obtain higher HR than the control group. For the total events, *I*^2^ test (*I*^2^ = 0% < 50%) indicated low heterogeneity (Fig. 11).

## Discussion

Stem cell therapy is an emerging treatment of perianal fistulas rooted the stem cells’ multipotential and self-renewal capacity [21, 34]. Stem cells have been categorised as embryonic or of adult-derived origin. However, due to the derivation of embryonic stem cells (ESCs), which creates ethical problems [35], ESCs were rarely applied in experiments. Adult-derived stem cells can be further categorised as haematopoietic stem cells, intestinal stem cells, MSCs, etc. [36, 37]. Among the stem cells, MSCs are the most administrated cells in fistulas treatment due to their ease of extraction.

This new application accounted for the following aspects. Firstly, MSCs promote the regeneration and migration of vascularity. Admittedly, angiogenic action is a key point in any type of damage healing for blood-carrying nutrients or metabolic waste products [38]. Moreover, the morbid state will influence the stem cells’ actions [39, 40], causing them to differentiate into endothelial cells to form vessels directly [41] and promote local cell migration and growth. Recently, protein-rich exosomes of stem cells were found, which could regulate vessel formation, such as VEGF-A [41, 42]. Secondly, they promote the proliferation and maturation of wound healing cells [43]. Wound healing relates to a variety of cells, including keratinocytes, fibroblasts, etc. Stem cells could also promote the process of those cells to repair the wound via the two ways mentioned above [44–47]. Finally, they downregulate immune responses and anti-inflammatory properties [41, 48–50]. Stem cells also regulate the over-expression of inflammatory mediators to protect normal tissues from oxidative damage and subsequent cytoclasis or apoptosis [43, 51, 52]. Therefore, MSCs are an alternative choice for complex perianal fistulas.

Recently, though many researchers have demonstrated the efficacy of MSCs therapy for complex perianal fistulas; however, to our knowledge, the study by Garcia-Olmo et al. [18] is the first report of an RCT in which stem cells obtained from mesenchymal tissues were used to treat perianal fistulas. To date, a number of deficient massive clinical trials of MSCs treatment are ongoing. Notably, two systematic reviews and meta-analyses have already been published on MSC treatment for perianal fistulas in 2019 and 2020 [8, 53], which were performed by the same one researching team and they analysed HR based on publications rather than clinical trials, so data from different publications were eligible to their analyses—but some data might associate with the same one clinical trial. In other words, the same group participants might be included twice even three times in their study. Our study corrected this flaw—we analysed data based on information within the clinical trials registry. In the study of Ciccocioppo et al. [54], both RCTs and one-arm clinical trials were included, while our study just included RCTs to increase accuracy.

Our study aimed to preliminarily distinguish the efficacy of MSCs therapy in different follow-up phases by adopting available data. Further, we aimed to discuss whether MSCs therapy could be considered a sort of therapy appending the positive effects in the short and long phases. To expand the sample quantity, absolute qualifications were not set for the basic demographics and clinical characteristics. For example, the participants of the eligible trials just needed to be diagnosed with complex fistulas, but the disease activity index, the course of diseases, or the number of fistulas were not required. As for intervention, significant heterogeneities existed in the studies concerning the cell dosage, different sources of MSCs, cell types and aetiologies, so we performed subgroup analyses appropriately to figure out whether those factors would influence treatment efficacy.

The results of Meta 1 (RR = 2.49; 95% CI 1.63, 3.80; *P* < 0.0001), Meta 3 (RR = 1.35, 95% CI 1.10, 1.67; *P* = 0.005), and Meta 4 (RR = 1.85, 95% CI 1.10, 3.10; *P* = 0.02) demonstrate that MSCs therapy for complex fistulas was effective in the short-term, long-term, and over-long-term follow-up phases and superior to conventional therapy (42.35% versus 18.81%; 55.96% versus 42.86%; 49.37% versus 26.00%). One important point to emphasise is that the longer the follow-up time, the more cases might withdraw. The follow-up results in the long-term and over-long-term follow-up phases might accompany high attrition bias, of which credibility was low. Figures 2 and 3 also display this condition—the long-term and over-long-term follow-up results in the studies by Hector [26], Barnhoorn [28], and Garcia-Olmo [31] were defined with attrition bias.

The result of Meta 2 (46.38% versus 37.88%; RR = 1.25; 95% CI 1.00, 1.55; *P* = 0.05) demonstrates no statistically significant difference; however, its P-value is in a critical state (*P* = 0.05). The HR of the MSCs group was superior to that of the control group in total events or in the most trials excepting clinical trial 5. Therefore, we deemed that MSCs therapy was more effective than traditional therapy in the medium-term follow-up phase after considering different aspects.

The afore-listed results demonstrated clearly that MSCs transplantation was an effective treatment method. Furthermore, apart from stem cells, adipose tissues have also been adopted for fistulas treatment in clinical trials [55, 56] because progenitor cells were contained. However, considering that the different treatment materials, MSCs and adipose tissues, might have different features, we did not include the trials in which tissues were used in the experimental group in our study.

When we compared the results of the six clinical trials—details are shown in Additional file 1: Figs. S7–S12 of Appendix 3—we observed increasing HR levels in the MSCs group over time in clinical trials 2, 3, and 4, which may be due to the characteristics of MSCs mentioned previously [8, 46, 57]. Among the trials, we observed unexpected outcomes in clinical trial 5—the HR of the MSCs group versus the control group was 30.43% versus 42.86% in 16 weeks follow-up phase and 55.00% versus 63.16% in 52 weeks follow-up. According to the protocol design, results, and discussion reported in the publication, in clinical trial 5, the investigators ‘corrected’ the design errors in previous phase II and phase III trials [18, 25, 26]—they established a clearer definition of ‘complex fistulas’, adopted stricter standards in operation and increased the cell dose to 100 million per injection [32]. The original purpose of the corrective measures was to reveal the efficacy of stem cell therapy under harsh terms; however, the reality was a reversal of the former trials in 16- and 52-week follow-up phase. Fortunately, the unexpected outcome between the two groups was not a statistically difference, which was not beyond expectations. Moreover, in the 2-year follow-up phase, the HR of the MSCs group was superior to that of the control group—a high number of over-long term recurrence were observed. Therefore, Garcia-Arranz et al. [32] concluded that ASCs for the treatment of perianal fistulas are safe and can improve long-term and sustained fistulas healing. This conclusion is also consistent with our study findings.

It is important to clarify why unexpected HR existed. The investigators thought these contrary result might associate with “cleaning surgery (deep curettage)” and “eliminating placebo effect (evaluating results by a surgeon blinded to treatment group)”. But in the former trails, blinding assessment also has been applied and its results were not as contrary as clinical trial 5. Thus, we think it might also be related to man factors, including the participants number, the dosage of MSCs, or other reasons, which has not been found. Although stem therapy for perianal fistulas is beneficial, many treatment details still require attention. We performed subgroup analyses concerning the cell dosage, different sources of MSCs, cell types and aetiologies based on the extracted data. In order to make more data could be included, we chose the HR of 1-year follow-up phase. Among six clinical trials, just clinical trial 3 lacked data of 1-year HR, and we chose 60-month following-up data to substitute.

In our study (Meta 5), MSCs therapy was effective for CD fistulas (57.36% versus 39.83%; RR = 1.43, 95% CI 1.09, 1.8; *Z* = 2.60, *P* = 0.009). But in cryptoglandular subgroup, the results of two trials displayed oppositely (clinical trials 2 and 5) and demonstrated no statistical significance. Few trails included were the limitation of our study, though this subgroup analysis could not offer enough evidence. We still tend to maintain that MSCs therapy was effective for cryptoglandular fistulas combining other clinical data—in a phase I trial, 11 out of 15 patients reaching radiographic improvement after MSCs treatment [58]—and the reason why clinical trial 5 displaying a reversal result were discussed before. In the future, more studies are needed to confirm this conclusion.

MSCs can be isolated from various tissues while adipose-derived and bone marrow-derived stem cells were the most common used types for fistulas treatment. Most studies concluded that whatever ASCs or BSCs were both effective for perianal complex fistulas. The results of meta 6 showed that both ASCs and BSCs could improve HR (55.96% versus 41.76%; 60.00% versus 33.33%). But there only one trail in BSCs group decreased the credibility of BSCs’ efficacy. Some studies supported that ASCs are equal even superior to BSCs in terms of immunosuppressive capacity [8, 59, 60]. Subcutaneous adipose tissue is a cradle of ACSs, and liposuction could help obtaining cells easily. However, obtaining BSCs is more difficult and dangerous for its extraction process is laborious and even the patients themselves fail to meet the extraction condition. In consideration of these aspects, ASCs might be recommended for treatment.

Excepting cell type, sources of MSCs are another arguing point, which concerning safety problems. In our study (Meta 7), autologous and allogenic MSCs could both improve HR compared with control (55.65% versus 45.68%; 56.78% versus 38.32%). In autologous subgroup, *Z* = 1.48, *P* = 0.14 indicated that no statistical difference existed in sub-analysis. This might cause by clinical trial 5. Combining with actual HR, we confirm that autologous MSCs were effective for fistulas as well as allogenic MSCs. Neoplastic developments were the main challenges in transplantation therapy, especially after finding that stem cells have relationships with cancer [61]. Given that the cell samples are obtained from the participants themselves in autologous subgroup, a neoplastic problem was not anticipated. In allogeneic subgroup, especially in clinical trial 3, one of the patients treated with 1 × 10^7^ MSCs developed adenocarcinoma of the cecum with peritoneal carcinomatosis more than 15 months after the intervention, and further evaluation revealed that this patient’s uncle died from colon cancer at the age of 42 years [27]. However, because a long period had elapsed between the procedure and adenocarcinoma development and a family history of cancer in this patient, we considered this event unrelated to the study treatment. In clinical trial 4, no abnormal tissue was formed, and so far, no finding of tumours emerging from MSCs therapy has been reported. Furthermore, in other disease studies, the safety of MSCs has already been confirmed in animal or clinical trials. In addition to perianal fistulas, MSCs therapy for the treatment of amyotrophic lateral sclerosis [62], heart failure [63], etc., has progressed into the clinical trial phase. Multiple applications of MSCs indicate its prospect in future medicine. Therefore, MSCs’ safety regarding neoplastic developments was admitted.

What’s more, adverse events (AEs) and serious adverse events (SAEs) were the common indicators for treatment safety, and AEs, although relatively common, were mostly minor. Comparing the eligible clinical trials, we observed that the criteria of AEs/SAEs were widely varied and difficult to compare. Though AEs/SAEs have not been summarised and analysed, we still consulted the full text of eligible publications to determine whether any AEs/SAEs were related to stem cell therapy, and it delighted us that no AEs/SAEs were directly related to MSCs therapy including the allogeneic MSCs groups. Also, no deaths occurred during the six trials. Overall, the more frequent AEs were proctalgia, abscess and pain and might have mainly been caused by the operation or the initial disease condition. This further proved safety of MSCs therapy.

The most suitable dosage of MSCs therapy for fistulas has not been defined. Garcia-Arranz et al. [32], the investigators of clinical trial 5, thought there was no evidence of a dose–response relation and their experimental results also proved this point. Meanwhile, the other investigators argued that there is a best-suited dosage for treatment and that a larger number of cells could behave immunogenic, resulting in increased clearance or deactivation of the cells [27, 64, 65]. In our sub-analysis, low dosage and high dosage MSCs can both improve the HR compared with control (55.56% versus 36.84%; 56.72% versus 43.51%). The pooled RR studies about low dosage MSCs therapy was 1.51 (95% CI 1.02, 2.21; *Z* = 2.08, *P* = 0.04) and about high dosage MSCs was RR = 1.30 (95% CI 1.02, 1.66). Based on eligible data, our results indicated that there was no difference of treatment efficacy concerning dosage change.

In addition to treat by MSCs or MSCs plus fibrin glue, some studies combined MSCs with other traditional medicine to treat fistulas. Knyazev et al. [66] performed a clinical trial to compare the effectiveness of combined therapy (local and systemic administration) of BSCs, BSCs (local administration), and certolizumab pegol (CZP) in CD fistulas. They demonstrated that combined cell and anti-cytokine therapy with CZP of CD with perianal lesions promotes more frequent and prolonged closure of simple fistulas, compared with BSCs monotherapy and CZP monotherapy. Knyazev et al. [67] performed a clinical trial to compare the effectiveness of combination therapy with BSCs, anticytokine therapy with infliximab (IFX), and antibiotic/immunosuppressive therapy in CD fistulas. They demonstrated that combined cellular and anticytokine therapy of CD with perianal lesions significantly contributes to the more frequent and prolonged closure of simple fistulas, as compared to antibiotics/immunosuppressors, and to a decrease in the frequency of recurrence of the disease. Those results showed that the combination of MSCs with other traditional medicine will have synergy effects. Because of the different mode of MSCs administration, so we did not include the combined therapy studies.

Although our study demonstrates the preliminary efficacy of MSCs therapy for fistulas, there are deficiencies in our study design. Therefore, more high-quality clinical trials are needed to supply current inferences. In addition, there are still many unsolved areas that require clarification: (1) if the best treatment dosage of MSCs exists; (2) whether the HR research should be based on the number of fistulas rather than patients; (3) unclear treatment mechanisms; (4) which kind of stem cells will work the best; and (5) the most appropriate technique for the MSCs extraction, conservation, and transplantation.

## Conclusions

Our study supports that MSCs transplantation could be a new therapeutic method for the treatment of complex perianal fistulas of two different origins with both short/long-term efficacy and sustained healing. The efficacy of MSCs of different cell types, cell sources and cell dosage is superior to the control. Besides, local MSCs therapy has shown more promising results of CD fistulas. Although we tend to maintain that MSCs therapy is effective for cryptoglandular fistulas equally, more studies are needed to confirm this conclusion in the future. There still remains unsolved questions, but the potential of MSCs is being gradually verified.

### Supplementary Information

**Additional file 1.** Materials to interpret for this study: search strategy of databases, the flow diagram of the six clinical trials and the HR of every clinical trial in different phases.

## Data Availability

All supporting data are included in the article and Additional file 1: Appendix 2.

## Abbreviations

CD: Crohn’s disease
MSCs: Mesenchymal stem cells
RCTs: Randomised controlled trials
HR: Healing rates
RR: Relative risk
CI: Confidence interval
ASCs: Adipose-derived MSCs
BSCs: Bone marrow-derived MSCs
ESCs: Embryonic stem cells
AEs: Adverse events
SAEs: Serious adverse events
CZP: Certolizumab pegol
IFX: Infliximab
