# Correction: Mesenchymal stem cells transplantation for perianal fistulas: a systematic review and meta-analysis of clinical trials

**DOI:** 10.1186/s13287-024-03664-w

**Published:** 2024-02-16

**Authors:** H. Wang, H. Y. Jiang, Y. X. Zhang, H. Y. Jin, B. Y. Fei, J. L. Jiang

**Affiliations:** 1 Scientific Research Center, China-Japan Union Hospital of Jilin University, Changchun, China; 2 Life Spring AKY Pharmaceuticals, Changchun, China; 3 Changchun University of Chinese Medicine, Changchun, China; 4 Department of Gastrointestinal Colorectal Surgery, China-Japan Union Hospital of Jilin University, Changchun, China

**Correction: Stem Cell Research & Therapy (2023) 14:103** 10.1186/s13287-023-03331-6

After the publication of this article, the authors noticed an error in the subheading of the Results section. The error was introduced during the revision process when the authors replaced the textual description in the subheading with symbols. The subheading “Meta 1: Efficacy of MSCs for complex perianal fistulas in short‑term follow‑up phase (≥ 3 months after treatment)” should be replaced by “Meta 1: Efficacy of MSCs for complex perianal fistulas in short‑term follow‑up phase (≤ 3 months after treatment)”.

The authors apologize for the error and confirm that the overall results and conclusions are not affected by the change.

