# Supplementary material for: Mesenchymal stem cells transplantation for perianal fistulas: a systematic review and meta-analysis of clinical trials
Source: Stem Cell Res Ther. 2023 Apr 26;14:103. doi: 10.1186/s13287-023-03331-6 (PMC10134595; doi:10.1186/s13287-023-03331-6)
Supplement: Supplementary file 1 — Additional file 1. Materials to interpret for this study: search strategy of databases, the flow diagram of the six clinical trials and the HR of every clinical trial in different phases. [file 13287_2023_3331_MOESM1_ESM.docx]

**Appendix 1 :**

**Search strategy of databases :**

1. Search strategy of PubMed :

((((("Rectal Fistula"[Mesh]) OR (((((((Fistula, Rectal[Title/Abstract]) OR (Anal Fistula[Title/Abstract])) OR (perianal Fistula[Title/Abstract])) OR (anorectal fistula[Title/Abstract])) OR (rectum fistula[Title/Abstract])) OR (fistula recti[Title/Abstract])) OR (fistula, rectum[Title/Abstract]))) OR (("Crohn Disease"[Mesh]) OR ((((((((((((((((((((Crohn's Enteritis[Title/Abstract]) OR (Regional Enteritis[Title/Abstract])) OR (Crohn's Disease[Title/Abstract])) OR (Crohns Disease[Title/Abstract])) OR (Inflammatory Bowel Disease 1[Title/Abstract])) OR (Enteritis, Granulomatous[Title/Abstract])) OR (Granulomatous Enteritis[Title/Abstract])) OR (Enteritis, Regional[Title/Abstract])) OR (Ileocolitis[Title/Abstract])) OR (Colitis, Granulomatous[Title/Abstract])) OR (Granulomatous Colitis[Title/Abstract])) OR (Ileitis, Terminal[Title/Abstract])) OR (Terminal Ileitis[Title/Abstract])) OR (Ileitis, Regional[Title/Abstract])) OR (Regional Ileitis[Title/Abstract])) OR (Crohn's perianal Fistula[Title/Abstract])) OR (enteritis regionalis[Title/Abstract])) OR (intestinal tract, regional enteritis[Title/Abstract])) OR (morbus crohn[Title/Abstract])) OR (regional enterocolitis[Title/Abstract])))) OR (("Inflammatory Bowel Diseases"[Mesh]) OR ((Inflammatory Bowel Disease[Title/Abstract]) OR (Bowel Diseases, Inflammatory[Title/Abstract])))) AND ((("Mesenchymal Stem Cells"[Mesh]) OR ((((((((((((((((((((((((((((((((((((((((((Stem Cell, Mesenchymal[Title/Abstract]) OR (Mesenchymal Stem Cell[Title/Abstract])) OR (Stem Cells, Mesenchymal[Title/Abstract])) OR (Bone Marrow Mesenchymal Stem Cells[Title/Abstract])) OR (Bone Marrow Mesenchymal Stem Cell[Title/Abstract])) OR (Bone Marrow Stromal Cells[Title/Abstract])) OR (Bone Marrow Stromal Cell[Title/Abstract])) OR (Bone Marrow Stromal Cells, Multipotent[Title/Abstract])) OR (Multipotent Bone Marrow Stromal Cell[Title/Abstract])) OR (Multipotent Bone Marrow Stromal Cells[Title/Abstract])) OR (Adipose-Derived Mesenchymal Stem Cells[Title/Abstract])) OR (Adipose Derived Mesenchymal Stem Cells[Title/Abstract])) OR (Adipose-Derived Mesenchymal Stromal Cells[Title/Abstract])) OR (Adipose Derived Mesenchymal Stromal Cells[Title/Abstract])) OR (Mesenchymal Stem Cells, Adipose-Derived[Title/Abstract])) OR (Mesenchymal Stem Cells, Adipose Derived[Title/Abstract])) OR (Adipose-Derived Mesenchymal Stem Cell[Title/Abstract])) OR (Adipose Derived Mesenchymal Stem Cell[Title/Abstract])) OR (Adipose Tissue-Derived Mesenchymal Stem Cell[Title/Abstract])) OR (Adipose Tissue Derived Mesenchymal Stem Cell[Title/Abstract])) OR (Adipose Tissue-Derived Mesenchymal Stem Cells[Title/Abstract])) OR (Adipose Tissue Derived Mesenchymal Stem Cells[Title/Abstract])) OR (Adipose Tissue-Derived Mesenchymal Stromal Cells[Title/Abstract])) OR (Adipose Tissue Derived Mesenchymal Stromal Cells[Title/Abstract])) OR (Adipose Tissue-Derived Mesenchymal Stromal Cell[Title/Abstract])) OR (Adipose Tissue Derived Mesenchymal Stromal Cell[Title/Abstract])) OR (Mesenchymal Stromal Cells[Title/Abstract])) OR (Mesenchymal Stromal Cell[Title/Abstract])) OR (Stromal Cell, Mesenchymal[Title/Abstract])) OR (Stromal Cells, Mesenchymal[Title/Abstract])) OR (Multipotent Mesenchymal Stromal Cells[Title/Abstract])) OR (Multipotent Mesenchymal Stromal Cell[Title/Abstract])) OR (Mesenchymal Stromal Cells, Multipotent[Title/Abstract])) OR (Mesenchymal Progenitor Cell[Title/Abstract])) OR (Mesenchymal Progenitor Cells[Title/Abstract])) OR (Progenitor Cell, Mesenchymal[Title/Abstract])) OR (Progenitor Cells, Mesenchymal[Title/Abstract])) OR (Wharton Jelly Cells[Title/Abstract])) OR (Wharton's Jelly Cells[Title/Abstract])) OR (Wharton's Jelly Cell[Title/Abstract])) OR (Whartons Jelly Cells[Title/Abstract])) OR (Bone Marrow Stromal Stem Cells[Title/Abstract]))) OR (("Stem Cells"[Mesh]) OR ((((((((((((((((Cell, Stem[Title/Abstract]) OR (Cells, Stem[Title/Abstract])) OR (Stem Cell[Title/Abstract])) OR (Progenitor Cells[Title/Abstract])) OR (Cell, Progenitor[Title/Abstract])) OR (Cells, Progenitor[Title/Abstract])) OR (Progenitor Cell[Title/Abstract])) OR (Mother Cells[Title/Abstract])) OR (Cell, Mother[Title/Abstract])) OR (Cells, Mother[Title/Abstract])) OR (Mother Cell[Title/Abstract])) OR (Colony-Forming Unit[Title/Abstract])) OR (Colony Forming Unit[Title/Abstract])) OR (Colony-Forming Units[Title/Abstract])) OR (Colony Forming Units[Title/Abstract])) OR (precursor cell[Title/Abstract]))))) AND (randomized controlled trial[Publication Type] OR randomized[Title/Abstract] OR placebo[Title/Abstract])

1. Search strategy of Embase :

1. Search strategy of Cochrane library database :

**Appendix 2 :**

Figures 1-6 show the flow diagram of the six clinical trials.

**
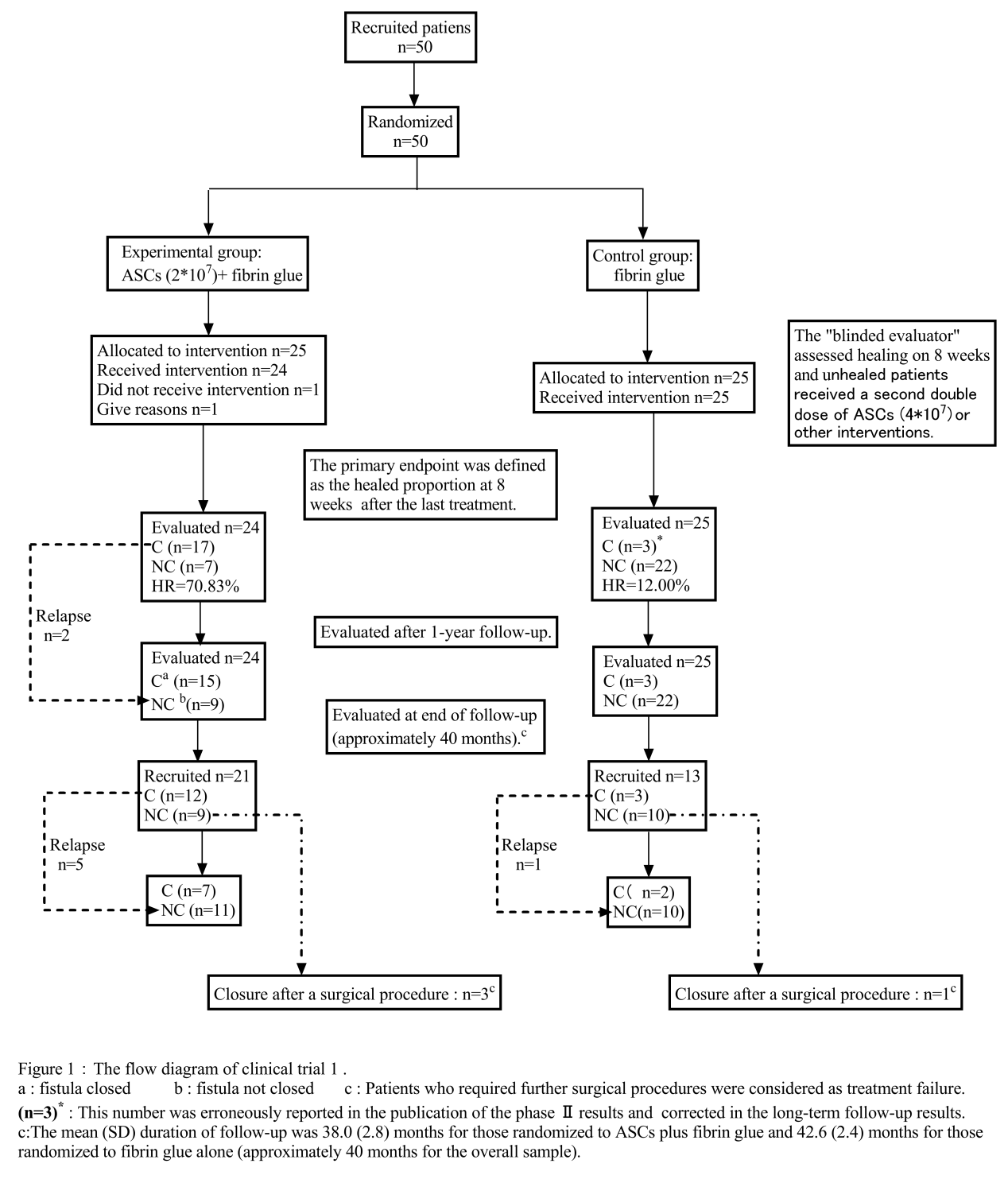

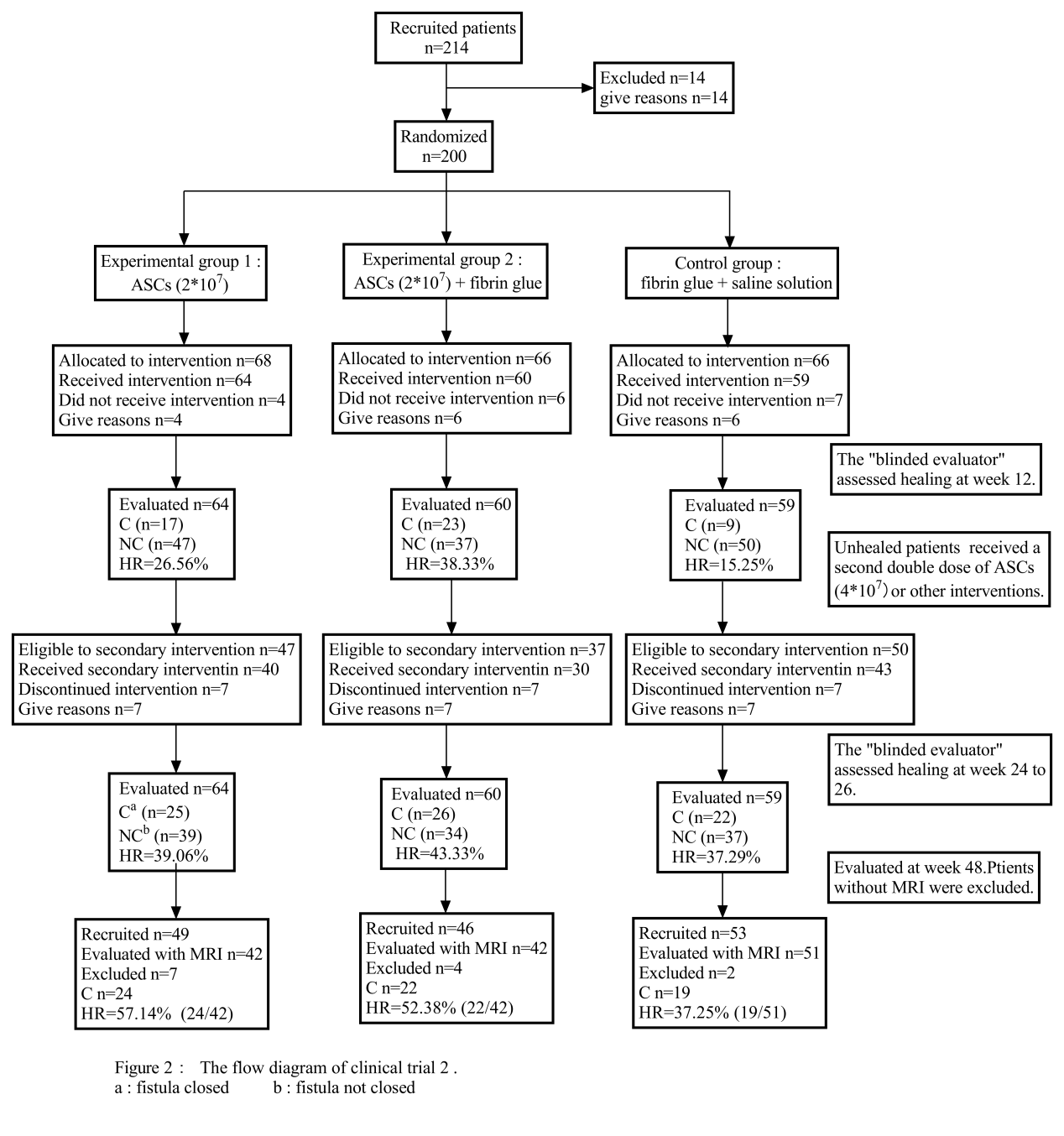

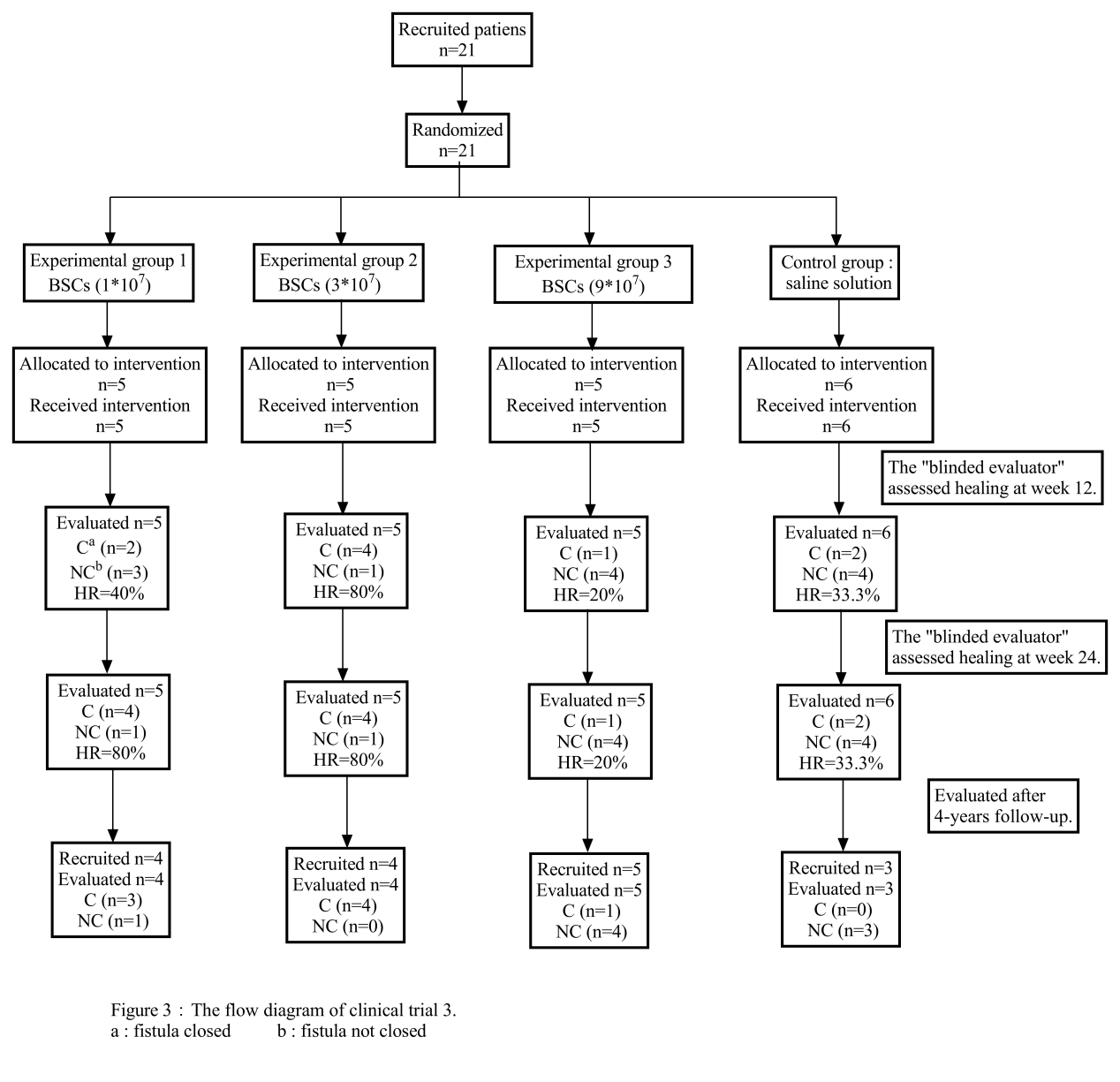

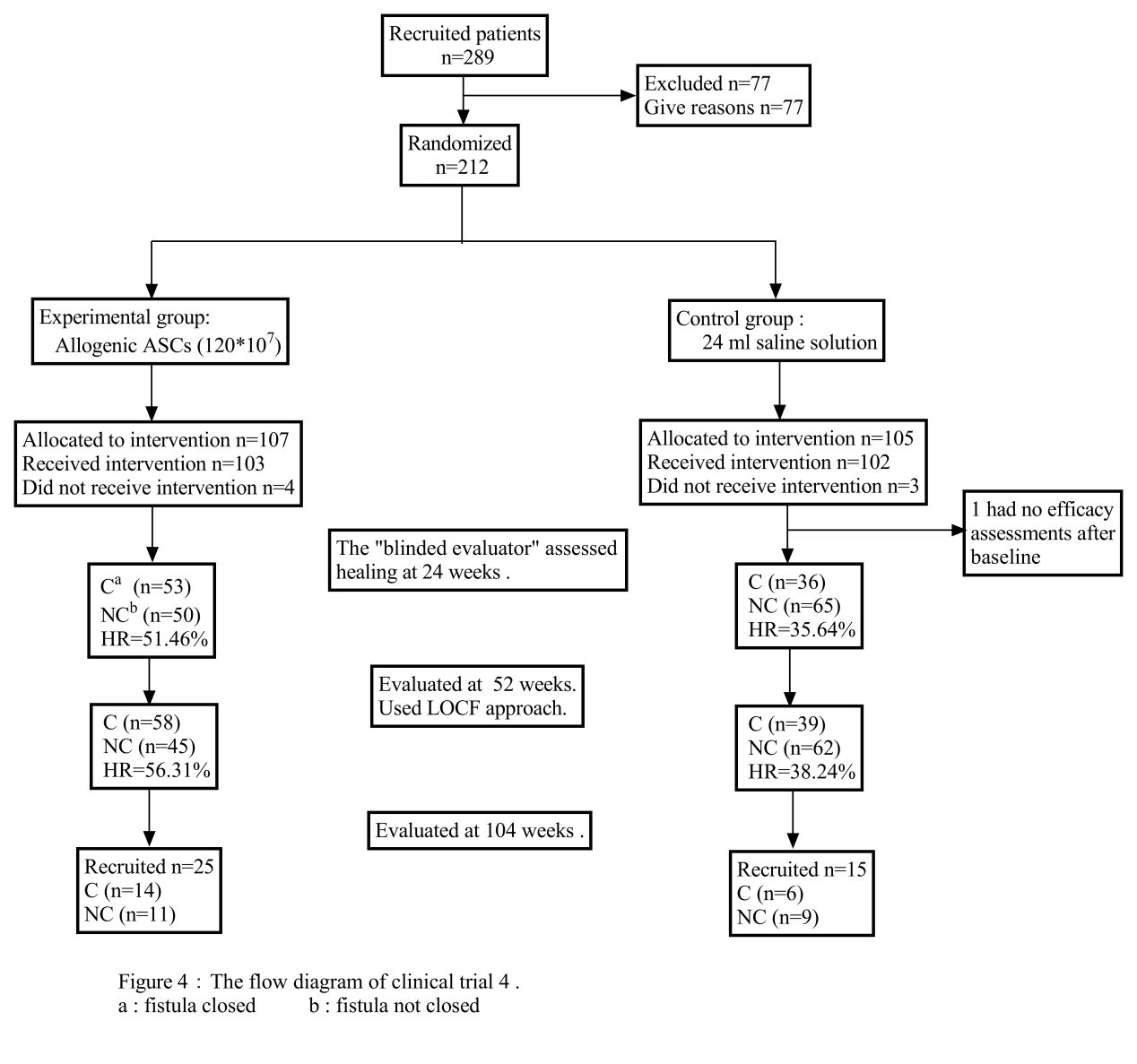

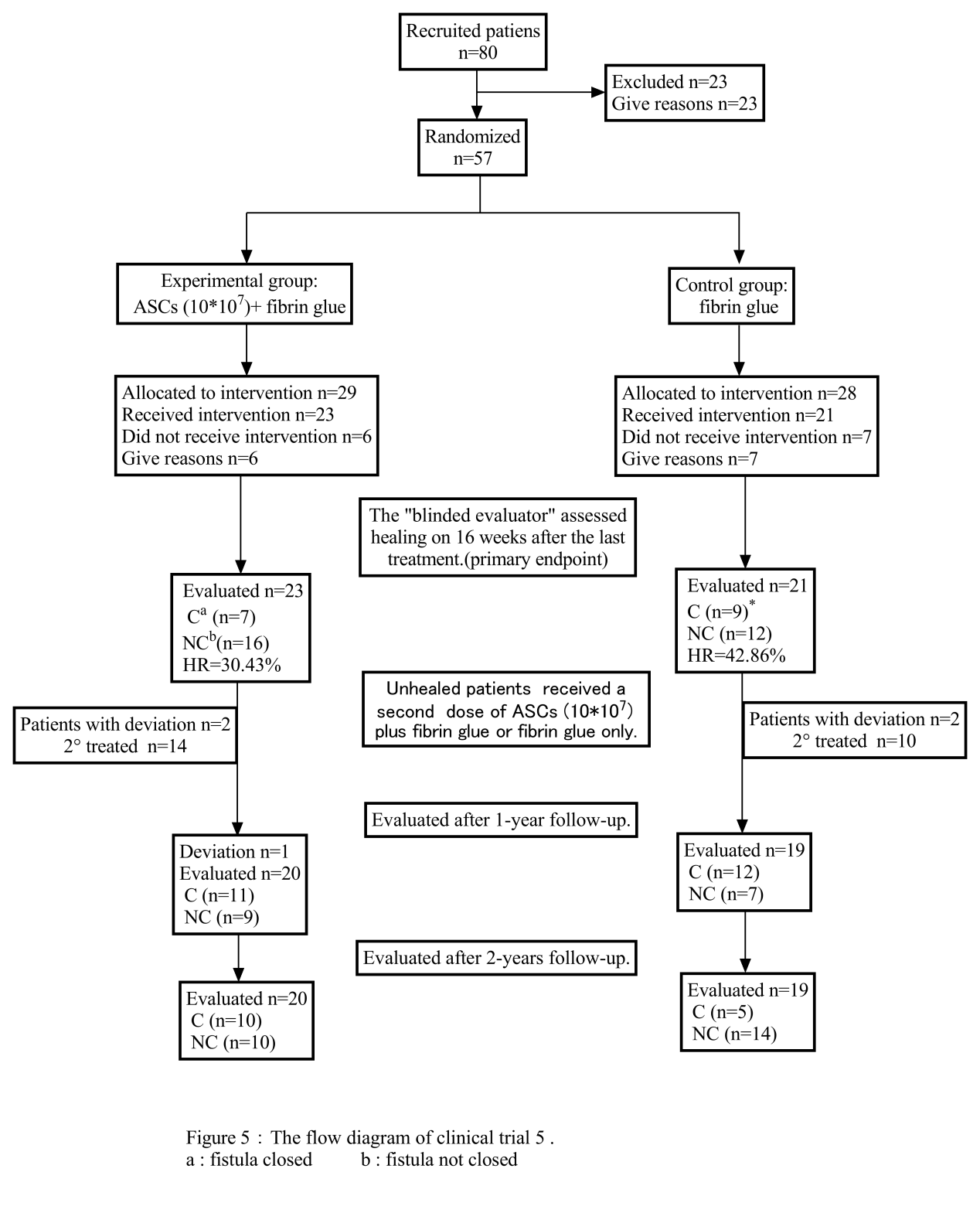

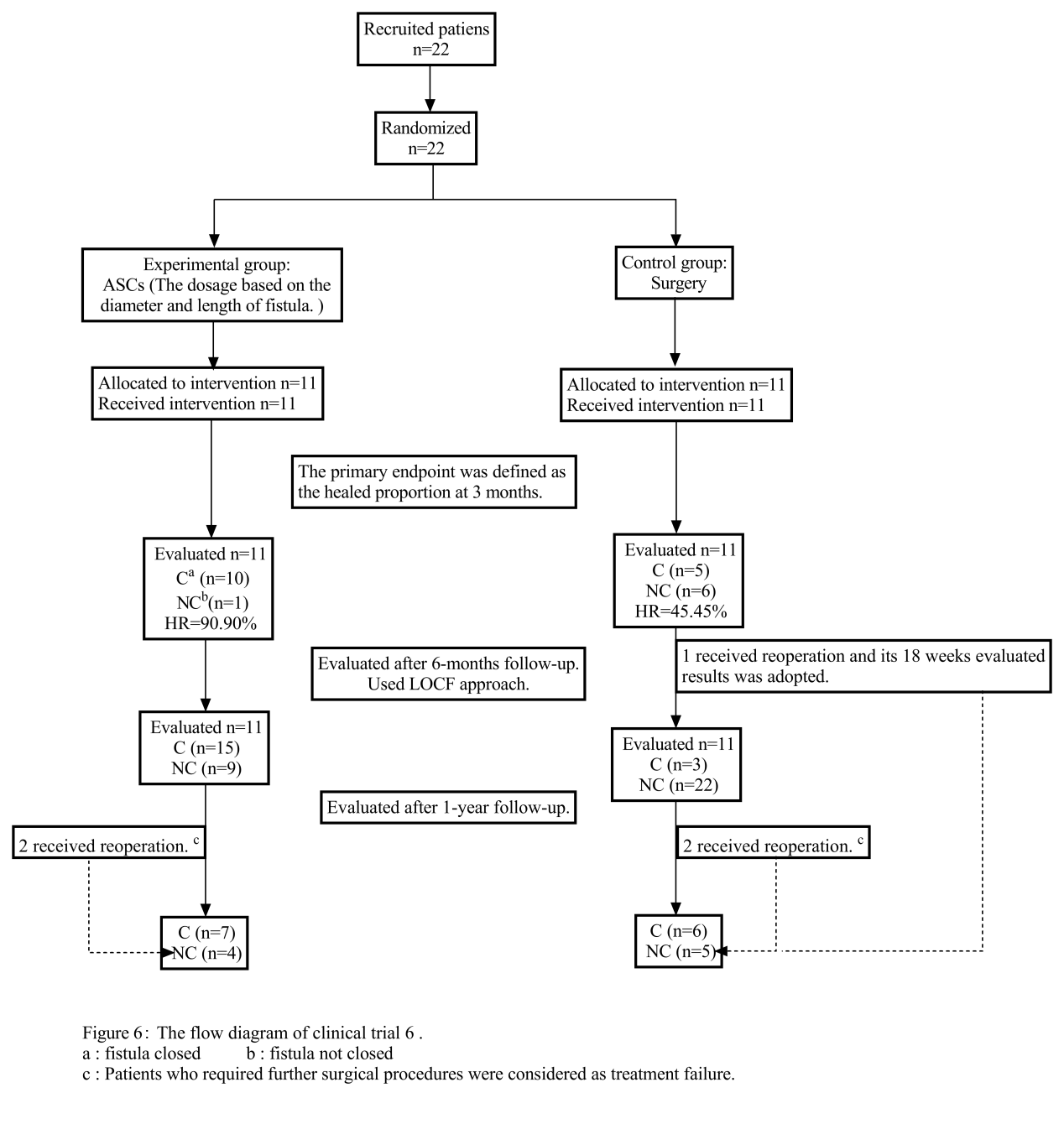
**

**Appendix 3 : The HR of every clinical trial in different phases**

Figures 7–12 show the HR of the six clinical trials in different follow-up phases. The trend indicated that the efficacy of MSCs was superior to that of traditional treatment for perianal fistula treatment. However, there was an exception in clinical trial 5, where the HR of the control group was higher than MSCs group in 16 weeks and 52 weeks follow-up phases. And the reason was discussed in “Discussion” part of this article.


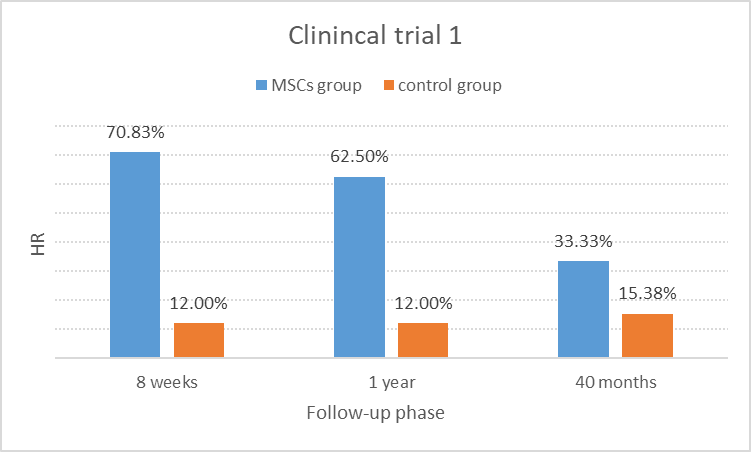


Figure 7 : The HR of clinical trial 1 in different follow-up phase


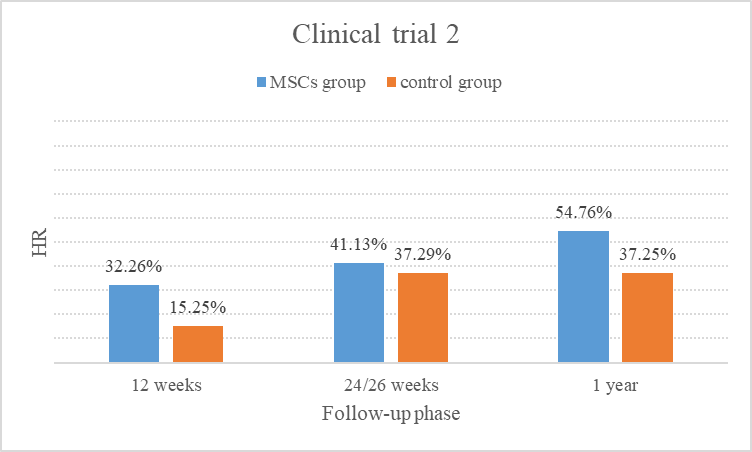


Figure 8 : The HR of clinical trial 2 in different follow-up phase


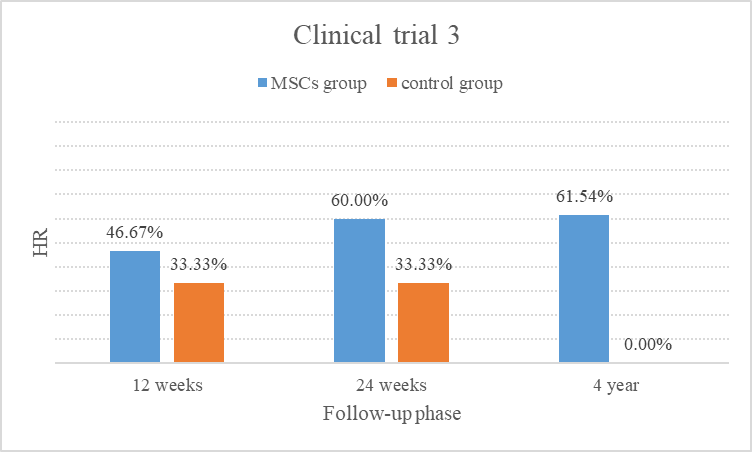


Figure 9 : The HR of clinical trial 3 in different follow-up phase


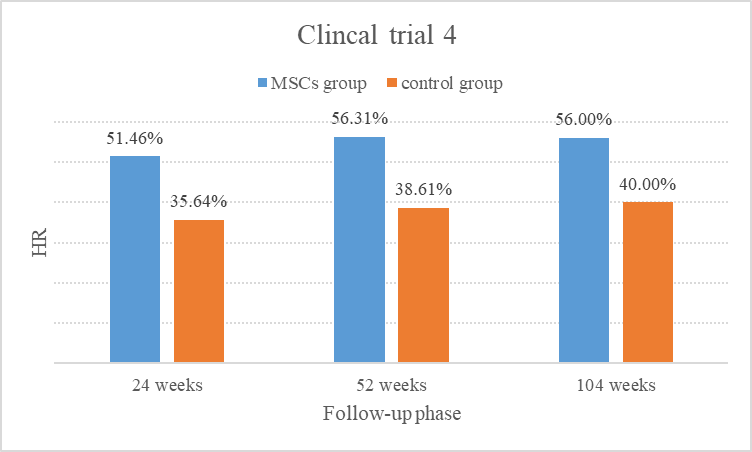


Figure 10 : The HR of clinical trial 4 in different follow-up phase


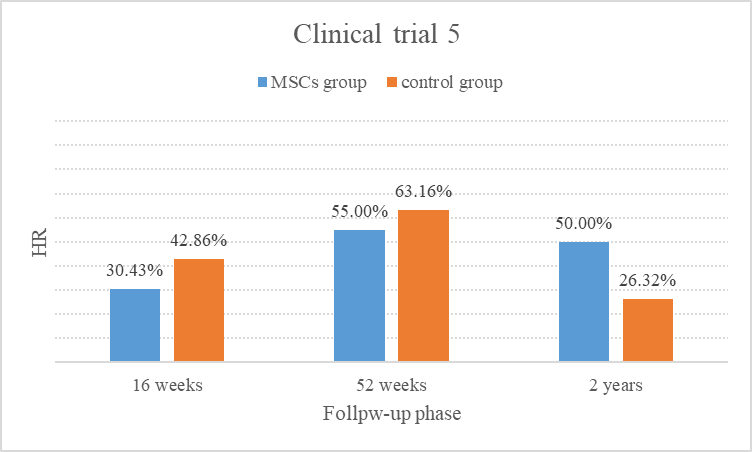


Figure 11 : The HR of clinical trial 5 in different follow-up phase


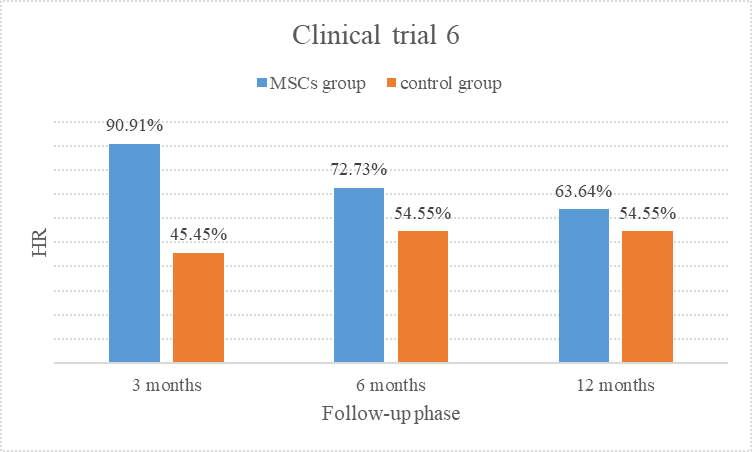


Figure 12 : The HR of clinical trial 6 in different follow-up phase
